# Transcription factor-dependent regulatory networks of sexual reproduction in *Fusarium graminearum*

**DOI:** 10.1128/mbio.03030-24

**Published:** 2024-11-26

**Authors:** Wonyong Kim, Da-Woon Kim, Zheng Wang, Meng Liu, Jeffrey P. Townsend, Frances Trail

**Affiliations:** 1 Department of Applied Biology, College of Agriculture and Life Sciences, Chonnam National University, Gwangju, South Korea; 2 Department of Plant Biology, Michigan State University, East Lansing, Michigan, USA; 3 Department of Biostatistics, Yale School of Public Health, New Haven, Connecticut, USA; 4 Department of Ecology and Evolutionary Biology, Yale University, New Haven, Connecticut, USA; 5 Department of Plant, Soil and Microbial Sciences, Michigan State University, East Lansing, Michigan, USA; Duke University, Durham, North Carolina, USA

**Keywords:** transcription factor, gene regulatory network, sexual development, perithecium, *Fusarium graminearum*

## Abstract

Transcription factors (TFs) involved in sexual reproduction in filamentous fungi have been characterized. However, we have little understanding of how these TFs synergize within regulatory networks resulting in sexual development. We investigated 13 TFs in *Fusarium graminearum*, whose knockouts exhibited abortive or arrested phenotypes during sexual development to elucidate the transcriptional regulatory cascade underlying the development of the sexual fruiting bodies. A Bayesian network of the TFs was inferred based on transcriptomic data from key stages of sexual development. We evaluated *in silico* knockout impacts to the networks of the developmental phenotypes among the TFs and guided knockout transcriptomics experiments to properly assess regulatory roles of genes with same developmental phenotypes. Additional transcriptome data were collected for the TF knockouts guided by the stage at which their phenotypes appeared and by the cognate *in silico* prediction. Global TF networks revealed that TFs within the mating-type locus (*MAT* genes) trigger a transcriptional cascade involving TFs that affected early stages of sexual development. Notably, *PNA1*, whose knockout mutants produced exceptionally small protoperithecia, was shown to be an upstream activator for *MAT* genes and several TFs essential for ascospore production. In addition, knockout mutants of *SUB1* produced excessive numbers of protoperithecia, wherein *MAT* genes and pheromone-related genes exhibited dysregulated expression. We conclude that *PNA1* and *SUB1* play central and suppressive roles in initiating sexual reproduction, respectively. This comprehensive investigation contributes to our understanding of the transcriptional framework governing the multicellular body plan during sexual reproduction in *F. graminearum*.

**IMPORTANCE:**

Understanding transcriptional regulation of sexual development is crucial to the elucidation of the complex reproductive biology in *Fusarium graminearum*. We performed gene knockouts on 13 transcription factors (TFs), demonstrating knockout phenotypes affecting distinct stages of sexual development. Using transcriptomic data across stages of sexual development, we inferred a Bayesian network of these TFs that guided experiments to assess the robustness of gene interactions using a systems biology approach. We discovered that the mating-type locus (*MAT* genes) initiates a transcriptional cascade, with *PNA1* identified as an upstream activator essential for early sexual development and ascospore production. Conversely, *SUB1* was found to play a suppressive role, with knockout mutants exhibiting excessive protoperithecia due to abnormally high expression of *MAT* and pheromone-related genes. These findings highlight the central roles of *PNA1* and *SUB1* in regulating other gene activity related to sexual reproduction, contributing to a deeper understanding of the mechanisms of the multiple TFs that regulate sexual development.

## INTRODUCTION

Filamentous fungi comprise a diverse group of organisms with profound ecological, industrial, medical, and agricultural significance. Sexual reproduction emerges as a fundamental mechanism fostering genetic diversity within populations. This diversity not only facilitates adaptation to challenging environments but also enables the colonization of new hosts in the case of pathogenic fungi, thus underscoring the importance of studying sexual reproduction in terms of the evolutionary dynamics of these organisms (1). The perithecium (pl. perithecia) is a flask-shaped reproductive organ produced as the outcome of mating (or selfing in homothallic fungi) in species belonging to the class Sordariomycetes. The production of perithecia also ensures effective dispersal of sexual spores (i.e., ascospores) via forcible spore discharge mechanisms (2). The intricate orchestration of gene expression during perithecium development is governed by a myriad of regulatory elements, such as transcription factors (TFs), protein kinases, and genes involved in the cellular signal transduction system (3). Sordariomycetes fungi, such as *Fusarium graminearum*, *Neurospora crassa*, *Podospora anserina*, and *Sordaria macrospora*, have served as model systems for studying the developmental processes associated with morphologically diverse perithecia (4–7). In particular, systematic genetic studies focusing on TFs, protein kinases, and phosphatases have been extensively conducted in *F. graminearum*, which revealed a number of regulatory factors crucial for perithecium development (8–10).

The two mating-type locus (*MAT*) genes, *MAT1-1-1* and *MAT1-2-1*, are arranged on the same chromosome and transcribed in a tail-to-tail configuration, with the 3´ regions of their transcripts significantly overlapping in *F. graminearum* (11). The *MAT1-1-1*, *MAT1-1-3*, and *MAT1-2-1* encode TFs containing a high-mobility group (HMG)-box domain and play pivotal roles in regulating the expression of many genes, including pheromone precursors and receptors (12–14). Despite approximately 100 TFs being implicated in sexual reproduction in *F. graminearum* (8), substantial knowledge gaps persist regarding their binding sequences and direct targets, with the exception of *MAT1-2-1* (14). Perithecium development involves a series of morphological and biochemical changes orchestrated by a complex regulatory network of TFs, which act in a cascade, initiating downstream growth and tissue differentiation (14–16). Post-mating responses include sequential development of sexual stage-specific tissues in *F. graminearum* (17). As protoperithecia develop, paraphyses (sterile hyphae) fill the central cavity of the growing fruiting body. Then, paraphyses recede, apparently undergoing autophagy, and the ascogenous system (fertile hyphae) expand, becoming the predominant tissue and producing asci (sac-like structures where ascospores form). In *F. graminearum*, development of new tissues, such as ascogonia (the female structures), protoperithecia, paraphyses, asci, and ascospores, takes place with approximately 1-day interval, making this model fungus ideal for studying time-course analysis of transcriptome data for knockouts showing developmental defects (18).

Sexual development from simple cellular aggregation to differentiation of multiple-tissue reproductive structure is considered being regulated by transcription factors likely form regulatory hubs (19, 20). Deletion of individual *MAT* genes, such as *MAT1-1-1*, *MAT1-1-2, MAT1-1-3*, and *MAT1-2-1*, caused aborted perithecium development, while knockouts of *MAT1-2-3* produced normal perithecia (13, 14). The roles of individual TFs were originally described in *N. crassa* and *S. macrospora*. In the heterothallic *N. crassa*, deletion of *asm-1* resulted in female sterility, but male fertility was retained (21). In the homothallic *F. graminearum*, knockouts lacking *StuA* (an ortholog of *asm-1*) did not develop perithecia and exhibited pleiotropic phenotypes with slower hyphal growth and delayed conidial production (22). Similar to *asm-1*, *adv-1* in *N. crassa* played crucial roles in sexual development, conidia production, and hyphal growth (5). In contrast, *Sordaria* strains lacking *pro1*, the homolog of *adv-1*, produce protoperithecia, suggesting its role played at later stages of female sexual development than that of *adv-1* (23). The *pro44* and *sub-1* are homologous TFs found in *S. macrospora* and *N. crassa*, respectively, whose knockouts lead to production of submerged protoperithecia in agar media (5, 24).

Understanding the contributions of TFs to regulatory networks is crucial for unraveling the molecular mechanisms underlying sexual reproduction, survival strategies, and spore dispersal in fungi. However, studies on gene regulatory networks in *F. graminearum* that consist of multiple TFs have been scarce (14, 25). Our previous studies demonstrate that some genes have a peak expression stage correlated with the developmental stage of their knockout phenotype (16, 26). However, knockouts of some TFs exhibit no obvious developmental phenotypes, and some exhibit similar phenotypes with perithecium development abortive or arrested at certain stages, making it hard to assess the regulatory orders directly with the knockout phenotypes. Here, we delineate the TF networks during sexual development in *F. graminearum* via multiple approaches, including construction of co-expression network, *in silico* simulation, and prediction of most informative next experiments. Data from previous transcriptomics experiments generated during sexual development were used to generate preliminary Bayesian co-expression networks and to infer systems biological models of the regulatory impacts. Phenotypes of selected TF knockouts were used to verify the models and interactions with other components of the regulatory network, revealing the possible interactive roles and orders of TFs along with developmental processes. This study seeks to pave the way for future research aimed at unraveling the intricate regulatory networks governing sexual reproduction in filamentous fungi.

## RESULTS

### Conserved transcription factors involved in perithecium development

**Fig 1:**
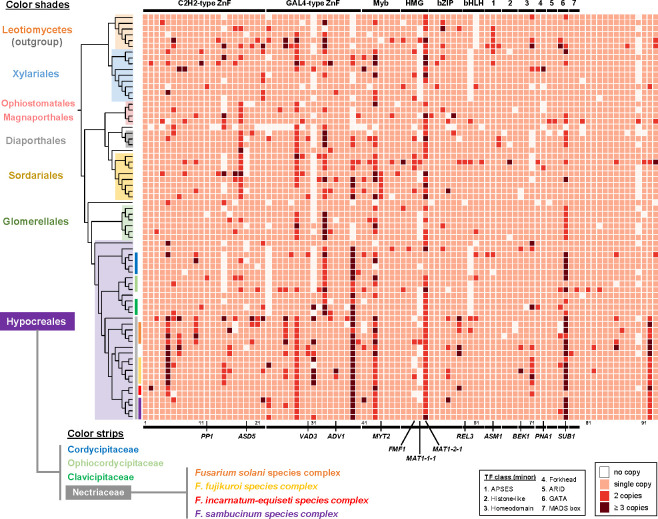
Phyletic distribution and conservation of transcription factors essential for sexual reproduction in fungi. The heatmap illustrates the presence and copy numbers of orthologous TF genes across 64 Sordariomycetes fungi and 6 Leotiomycetes fungi (used as an outgroup), focusing on 93 TFs functionally characterized in *F. graminearum*. Genomic data were obtained from the NCBI reference sequence database and analyzed using the OrthoFinder program. TF families are denoted above the heatmap, while the 13 TFs specifically investigated in this study are labeled below. The phylogenetic relationship among 70 species was inferred using STRIDE, with *F. graminearum* positioned in the bottommost leaf in the phylogenetic tree. Additional taxonomic information for 70 species is provided in Table S1.

Based on a previous high-throughput knockout study and other recent publications (8, 14, 27–39), we identified 93 TF genes as having knockout phenotypes affecting sexual reproduction in *F. graminearum*, many of which exhibit significant developmental stage-specific regulatory dynamics in their expression (Table S1; Fig. S1). Of the 93 TFs, the most common family was the C_2_H_2_-type TF family, followed by the GAL4, Myb (myeloblastosis), HMG-box, bZIP (basic leucine zipper), and bHLH (basic helix-loop-helix) families (Fig. 1). Several other TF families were also found to affect perithecium development in *F. graminearum*.

To assess the conservation of these 93 TFs, which are crucial for sexual reproduction *in F. graminearum*, we identified orthologous genes in the genomes of diverse fungi encompassing major orders within the class Sordariomycetes: *Fusarium* and *Trichoderma* spp. from the Hypocreales, *Colletotrichum* spp. from the Glomerellales, *N. crassa* and *S. macrospora* from the Sordariales, and *Xylaria* and *Hypoxylon* spp. from the Xylariales. TF families were missing in some taxa, while other TF families exhibited copy-number variations (Fig. 1). Notably, copy numbers for the orthogroups containing *MAT1-2-1* and *SUB1* were variable among the Sordariomycetes fungi. The orthogroup including TFs with an HMG-box consists of three TFs, *MAT1-2-1*, *MAT1-1-3*, and FGRRES_05151 (an ortholog of *PaHMG8*), in the homothallic fungus *F. graminearum*, while heterothallic fungi have either *MAT1-2-1* or *MAT1-1-3*. The presence of a *PaHMG8* homolog was variable among the Sordariomycetes fungi. The orthogroup including *SUB1* exhibited two additional putative TF containing a GATA domain, especially in the Hypocreales. The two novel GATA factors were not homologous to previously characterized GATA factors identified in *Aspergillus nidulans*, such as *areA*, *sreA*, and *wc-2*. Nevertheless, the majority of the TF families important for sexual reproduction are present as single-copy orthologs in Sordariomycetes. Orthologous genes of the 93 TFs are present in species closely related to *F. graminearum*, namely the *Fusarium sambucinum* species complex, with the exception of the idiomorphic gene *MAT1-1-1*, which is absent in a strain of *Fusarium poae* (Fig. 1). With the exception of a bHLH domain TF family, orthologs of all the TF families also can be found in the genomes of species belonging to the Leotiomycetes, which is sister to the Sordariomycetes.

### Stage-specific roles of transcription factors in perithecium development

**Fig 2:**
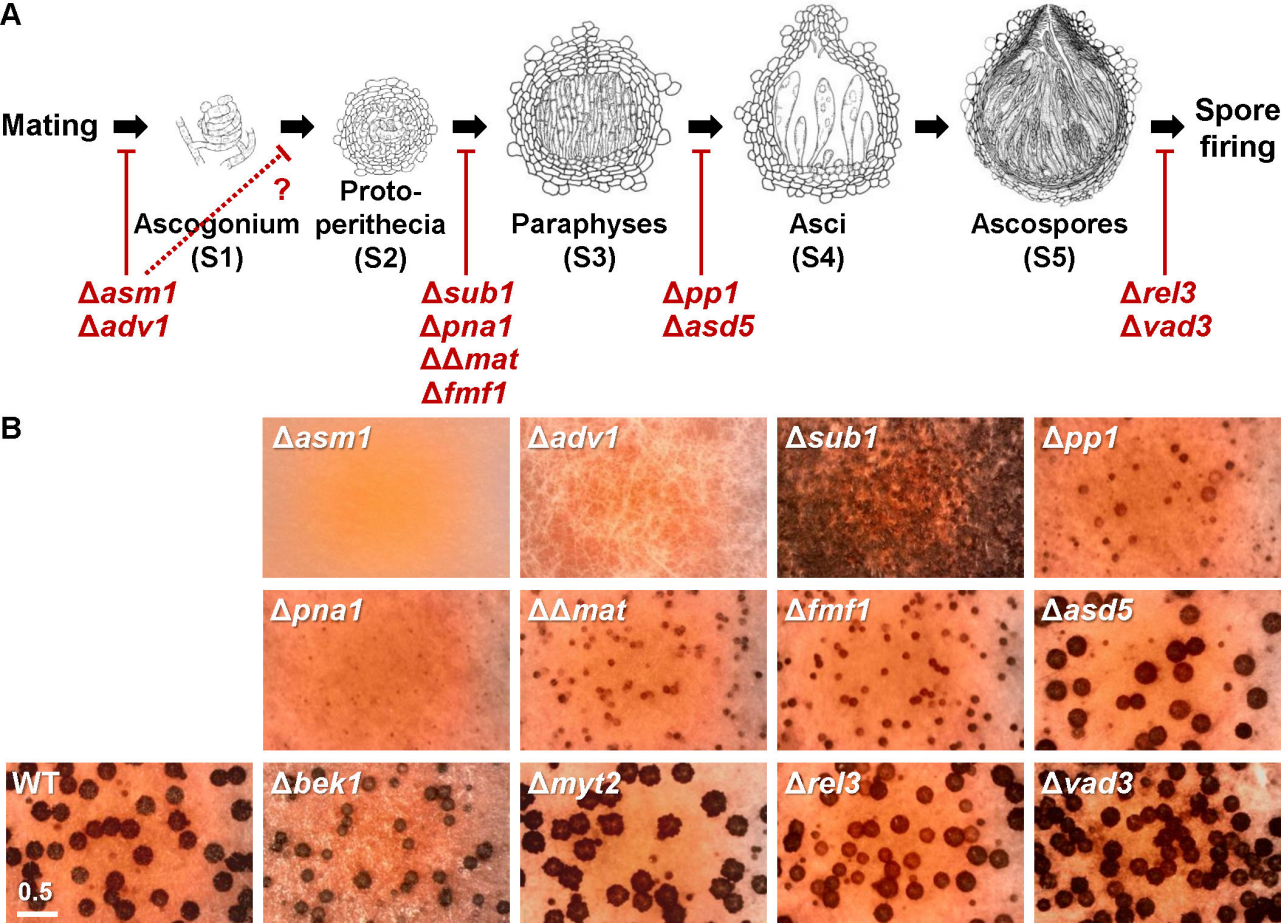
Phenotypic defects observed in mutants of the selected transcription factors. (**A**) Developmental stages of the perithecium in *F. graminearum*. Stage 1: formation of ascogonium (perithecium initial); stage 2: developing young perithecium (protoperithecium), comprised of central ascogenous cells. Stage 3: paraphyses filling in the central cavity of perithecium; stage 4: asci develop as the paraphyses senesce; stage 5: formation of mature ascospores. Cartoons were adapted from Kim et al. (41), not drawn to scale. Red blunted arrows indicate the stages at which TF knockouts affect the sexual phenotypes in *F. graminearum*. (**B**) Defective phenotypes of TF knockouts grown on carrot agar medium. Photos were taken 7 days after sexual induction. Scale bar: 500 µm. Detailed microscopy photos showing defective phenotypes of TF knockouts are provided in Fig. S2.

To understand the transcriptional cascade and regulatory network, we selected TFs that have been shown to have evolutionarily conserved function with those in *N. crassa* during sexual development (19) (Table 1). We also chose TFs whose knockout affected different stages of perithecium development, based on the previous observations in *F. graminearum* strains GZ3639 and GZ3643 (8, 14) (Fig. 2A). We generated knockout transformants for the selected TFs in the NCBI reference strain PH-1. In addition, we obtained a knockout strain lacking *FgStuA* (an ortholog of *asm-1* in *N. crassa*) (40). In this study, gene names follow orthologs in the genome of *N. crassa*, the model organism for studying Sordariomycetes fungi (e.g., *ASM1* instead of *StuA*). The two HMG-box domain TFs, *MAT1-1-1* and *MAT1-2-1,* in the mating-type locus play pivotal roles in the sexual reproduction in *F. graminearum* (11, 12, 14). Therefore, we generated a double-knockout strain lacking both *MAT1-1-1* and *MAT1-2-1* (here referred as ΔΔ*mat*).

**TABLE 1:** Transcription factors selected in this study

No.	Gene ID	Gene name	TF family	Knockout phenotype
1	FGRRES_01307	*REL3*	bHLH domain	Normal perithecia, but no firing
2	FGRRES_01366	*FMF1*	HMG-box domain	Protoperithecia only
3	FGRRES_05475	*BEK1*	Homeobox domain	Normal perithecia, but smaller
4	FGRRES_05503	*VAD3*	GAL4-type zinc finger	Normal perithecia, but no firing
5	FGRRES_07067	*ADV1* ^*a*^	GAL4-type zinc finger	No perithecia
6	FGRRES_07310	*PP1* ^*b*^	C_2_H_2_-type zinc finger	Protoperithecia only
7	FGRRES_07546	*MYT2*	Myb family	Normal perithecia, but bigger
8	FGRRES_08892	*MAT1-1-1*	HMG-box domain	Protoperithecia only
9	FGRRES_08893	*MAT1-2-1*	HMG-box domain	Protoperithecia only
10	FGRRES_10129	*ASM1* ^*c*^	APSES domain	No perithecia
11	FGRRES_15782	*PNA1*	Forkhead-box domain	Protoperithecia only
12	FGRRES_16570	*ASD5*	C_2_H_2_-type zinc finger	Immature asci and no ascospore
13	FGRRES_17300	*SUB1* ^*d*^	GATA-type zinc finger	Profuse protoperithecia

^*a*^ The ortholog of *pro1* in *Sordaria macrospora.*

^*b*^ The ortholog of *steA* in *Aspergillus nidulans*; and *MST12* in *Magnaporthe oryzae.*

^*c*^ The ortholog of *stuA* in *A. nidulans*.

^*d*^ The ortholog of *pro44* in *S. macrospora*; and *nsdD* in *A. nidulans.*

A total of 12 TF knockouts were examined for phenotypic defects during vegetative growth and sexual reproduction in detail (Fig. 2B; Fig. S2). In brief, Δ*asm1* and Δ*adv1* failed to develop protoperithecia, as previously reported (8, 22). Many knockouts, such as Δ*fmf1*, ΔΔ*mat*, Δ*pna1*, and Δ*sub1*, were arrested at the protoperithecium stage, and were not able to form viable ascospores (Fig. S2A). It is noteworthy that Δ*sub1* produced a profusion of submerged protoperithecia, especially when treated with 2.5% Tween 60 for sexual induction (Fig. S2B). Croziers, immature asci, and ascospores were observed (albeit infrequently) in squashed mounts of protoperithecia of Δ*pp1* (Fig. S2C). Production of paraphyses and asci were evident in Δ*asd5* (*as*cus development 5, first designated in this study); however, asci were immature, and no ascospores were observed in the perithecia (Fig. S2C). The defective phenotypes of Δ*rel3* and Δ*vad3* were similar; these deletions each resulted in a significant reduction of spore discharge at 7 days after sexual induction (Fig. S2D). Our Δ*bek1* strain produced visually normal asci and ascospores; however, the perithecium size was significantly smaller than the wild-type (Fig. 2B; Fig. S2E). To investigate the genetic factors determining perithecium size, we also included Δ*myt2* that produced large and bumpy perithecia (Fig. 2B) (42). As revealed in a previous study (8, 40), hyphal growth and conidiation were all normal in the TF knockouts with the exception of Δ*asm1.*

### Assessing impacts of transcription factors in a Bayesian network (BN)

**Fig 3:**
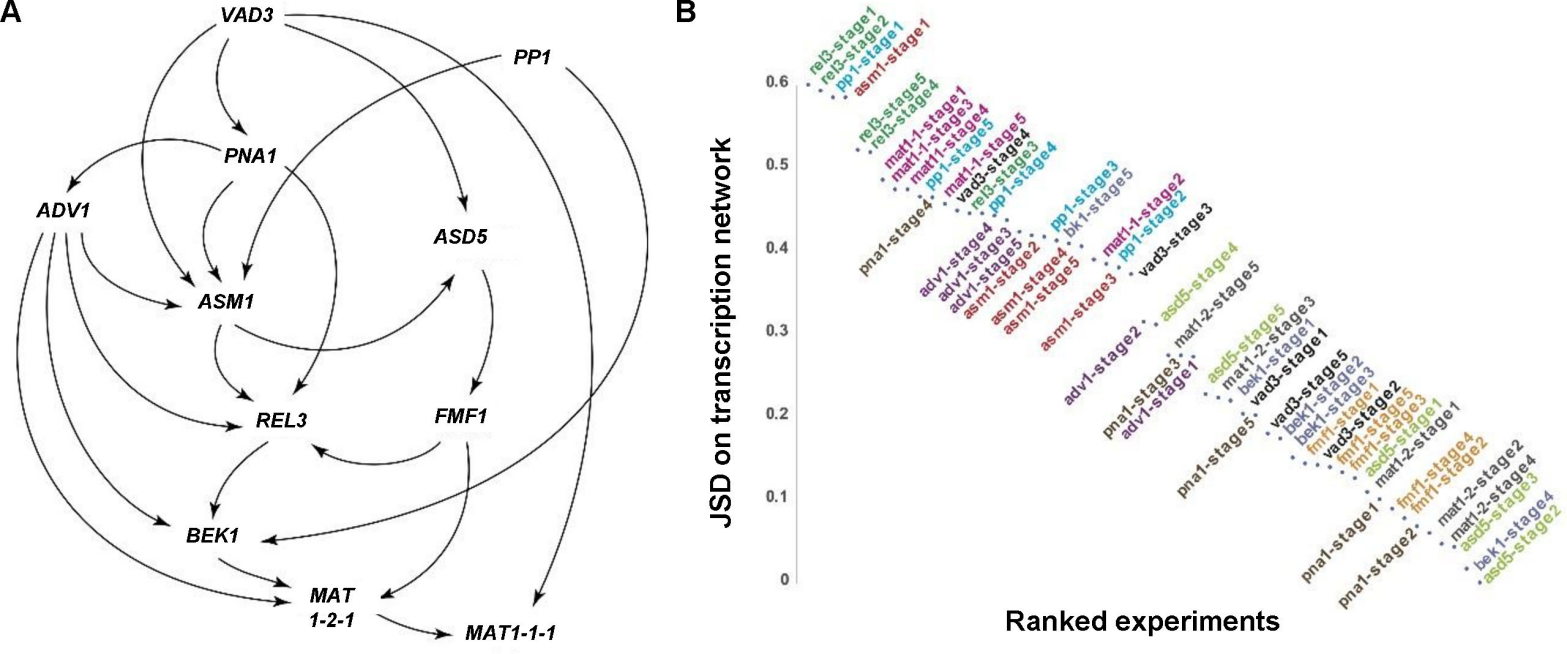
Bayesian network of transcription factors and application of the Perturbation to 0 to Predict Correlated Network Solidity (P0PCorNS) criterion. (**A**) Bayesian network of TFs during sexual development of *F. graminearum*. Fold-changes of TF gene-expression levels were sampled from six stages of sexual development and analyzed using the Bayesian Network Webserver. Regulatory edges are supported; directions of arrows may not correspond with direction of regulation. (**B**) Impacts of TFs were inferred with the SMILE with the JSD calculated for Bayesian networks based on the *in silico* knockout expression profiles. Y-axis: JSDs between predicted *in silico* BNs and the original BNs. Gene-stage JSDs indicating the informativeness of assessment of gene expression in the gene at the indicated developmental stage were ranked from high to low with individual genes color coded.

We constructed BNs to assess possible regulatory interactions among the TFs using the fold-change gene-expression data collected from six key stages of the sexual development (41). BNs of 11 TFs were investigated using Bayesian Network Webserver (43), excluding *SUB1* whose knockout caused profuse production of perithecial initials. The network positioned *PNA1*, *ASM1*, *ADV1*, and *REL3* forming a central regulatory hub with the mating-type loci genes, and having *PP1* and *VAD3* being more peripheral (Fig. 3A). The edges of BN were not interpreted with regulatory orders or direction, and peripheral nodes could be associated with either upstream or downstream roles related to the central hub. We further applied a Perturbation to 0 to Predict Correlated Network Solidity (P0PCorNS) criterion to quantify stage-specific impacts on the predicted BNs for TF knockouts through an *in silico* gene knockout approach. Stage-specific impacts of TFs were inferred and ranked based on the Jensen-Shannon Divergence (JSD) that measures the differences among the *in silico* stage-specific knockout networks with the predicted TF regulatory network. *In silico* knockout at any stage for a gene with essential regulatory roles in the process would exhibit high impacts to the network, and the stage with the highest impacts would be considered as the peak of the regulatory function of the gene and prioritized for transcriptomics experiments to assess the genome-wide impacts of the gene. Stage-specific impacts of *ADV1*, *ASM1*, *MAT1-1-1*, *PP1*, and *REL3* were aggregated and positioned high in the rank (Fig. 3B), suggesting their essential roles in the regulatory hub were robust. Impacts of *PNA1* and *VAD3* were also highly ranked high at some later stages, but the ranking of the informativeness to the TF network of their experimental knockout varied at other stages.

### Generation of transcriptome data for transcription factor knockouts

The phenotypic defects observed in TF knockouts provided crucial insight into functional roles of these TFs. To further investigate interactions among TFs—particularly those associated with genes exhibiting similar mutant phenotypes in perithecium development—analysis of transcriptomes of the TF knockouts was performed with regulatory network modeling. We obtained TF-knockout transcriptome data at developmental stages in which the mutants exhibited defective phenotypes. For example, we selected stage 1 for Δ*sub1*, as the mutant produced a higher number of submerged protoperithecia, which is indicative of an increased formation of ascogonia. We selected stage 3 for Δ*myt2*, which produced larger perithecia, to compare the RNA-seq data with that of Δ*bek1*, which produced smaller perithecia. Additionally, we opted for developmental stages for which the greatest impacts were predicted in a TF knockout using the P0PCorNS criterion. For examples, we selected stage 1 for Δ*rel3* and ΔΔ*mat*, in which *in silico* analysis of the TF gene perturbation led to the greatest contraction of the ensemble of permissible BNs (Fig. 3B).

**Fig 4:**
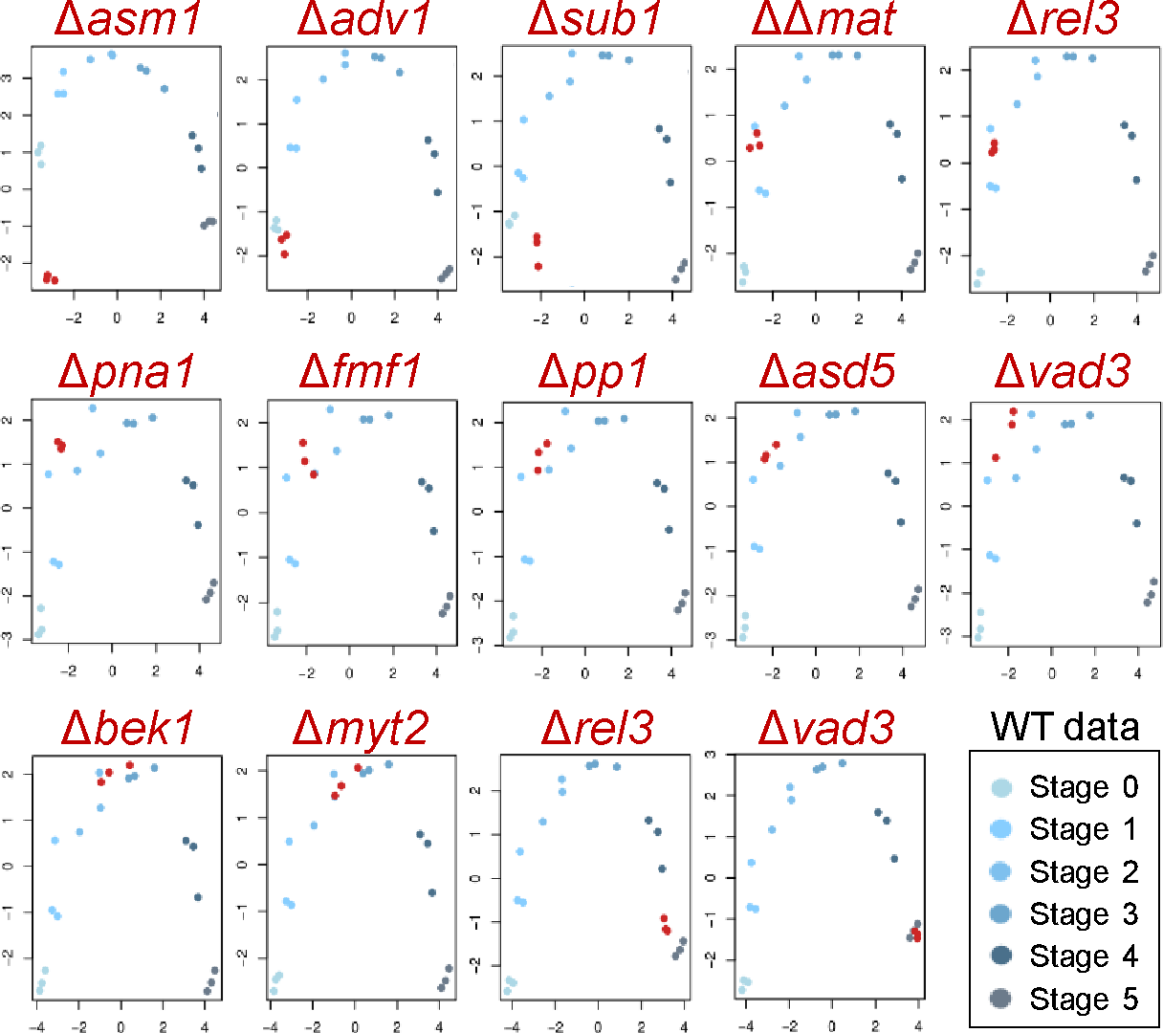
Multidimensional scaling (MDS) plots of RNA-seq data. Transcriptome data for transcription factor knockouts (red circles) and the wild-type data during sexual development (stage 0–stage 5; see the inset in the bottom) were plotted, based on their gene-expression profiles. Each point corresponds to an individual sample, with the distance between points reflecting the dissimilarity in gene-expression patterns. The plot illustrates that samples within the same developmental stage cluster together. The x-axis (the first dimension) and y-axis (the second dimension) are the two dimensions that best preserve the pairwise dissimilarities among the samples, capturing the major sources of variation in the data.

The resulting RNA-seq data for TF knockouts mainly corresponded to the wild-type data set at the same developmental stage, consistent with the notion that a TF controls only a subset of genes and does not substantially affect the majority of genes (Fig. 4). However, the RNA-seq data suggested that knockouts of some TFs, including Δ*adv1*, Δ*asm1*, Δ*bek1*, Δ*pp1*, and Δ*sub1*, resulted in retardation of perithecium development compared to development of the wild-type at the same timepoint after sexual induction. The transcriptome data of Δ*asm1*, sampled at stage 1, exhibited significant deviation from the wild-type transcriptome, reflecting its pleiotropic phenotypes on both vegetative and reproductive growth.

### Biological processes controlled by the selected TFs

**Fig 5:**
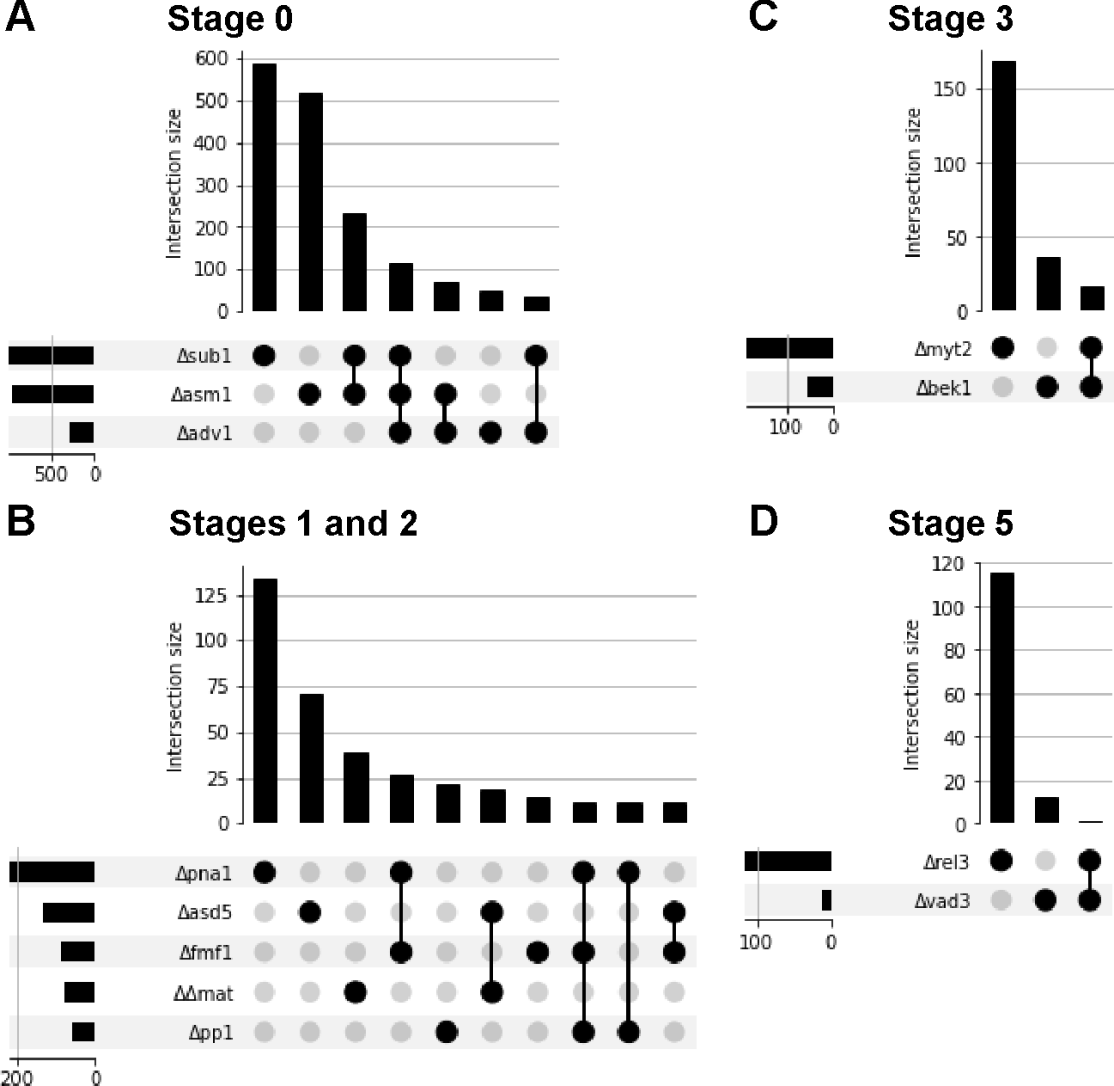
UpSet plots of differential gene-expression analyses. (A–D) Graphs on the lower left side of each panel display the total number of DE genes (x-axis) in transcription factor knockouts (y-axis). The bar graphs above in each panel exhibit the intersection of sets of DE genes (y-axis) shared between pairs of knockouts (x-axis), or the number of DE genes with no intersection.

We conducted differential gene-expression analyses using transcriptome data from the TF knockouts, comparing them with developmentally matched data from the wild-type (Fig. 4). At stage 0 (2 h after sexual induction), several hundred differentially expressed (DE) genes were identified in Δ*asm1* and Δ*sub1* (the complete list of DE genes are provided in Table S2). A substantial proportion of DE genes observed in Δ*adv1* were shared between Δ*asm1* and Δ*sub1*. However, the expression levels of many DE genes were specifically influenced by the deletion of *ASM1* or *SUB1* (Fig. 5A). In these knockouts, gene ontology (GO) terms such as translation (GO: 0006412) and proteolysis (GO: 0006508) were significantly enriched, suggesting the crucial role of these TFs in the regulation of protein metabolism during the initiation of sexual reproduction (Table 2). Among the TF knockouts displaying defective phenotypes in protoperithecium and ascospores formation, Δ*pna1* exhibited the highest number of DE genes at stage 2 (Fig. 5B). Although a substantial proportion of DE genes were shared between Δ*pna1*, Δ*pp1,* and Δ*fmf1*, DE genes observed in Δ*asd5*, Δ*pna1*, Δ*pp1*, and ΔΔ*mat* were mostly unique, indicating distinct regulatory roles of these TFs during protoperithecium development (Fig. 5B). Functional enrichment analysis highlighted the genes related to carbohydrate metabolism, so-called CAZymes, in Δ*pna1*, suggesting the role of the *PNA1*, a folkhead domain-containing TF, in transition of nutrient utilization for protoperithecium formation (Table 2).

**TABLE 2:** Functional enrichment analysis of differentially expressed genes

Knockout	GO number	GO category^*a*^ (biological process)	Adjusted *P*-value	DE genes (no. of genes in category)
Δ*asm1* at stage 0	GO:0006412	Translation	1.11e−11	42 (228)
	GO:0005975	Carbohydrate metabolic process	1.86e−05	66 (637)
	GO:0006979	Response to oxidative stress	1.55e−04	6 (15)
	GO:0006508	Proteolysis	4.28e−04	23 (172)
Δ*sub1* at stage 0	GO:0006412	Translation	1.49e−05	30 (228)
	GO:0006508	Proteolysis	1.97e−04	23 (172)
Δ*adv1* at stage 0	GO:0048856	Anatomical structure development	1.95e−05	3 (4)
	GO:0006508	Proteolysis	8.74e−05	12 (172)
Δ*pna1* at stage 2	GO:0005975	Carbohydrate metabolic process	2.09e−05	22 (637)
	GO:0046373	L-arabinose metabolic process	2.13e−05	3 (5)
Δ*rel3* at stage 5	GO:0006811	Monoatomic ion transport	6.07e−06	3 (7)

^*a*^ GO lists with adjusted *P*-value smaller than 5.00E−02 were reported.

We aimed to contrast the transcriptome data between Δ*bek1* and Δ*myt2*, as the TF knockouts exhibited opposing phenotypes; the former produced smaller perithecia, and the latter produced larger perithecia, compared to the wild-type. There was no functional enrichment in DE genes identified in Δ*bek1* and Δ*myt2* at stage 3. However, we identified several CAZymes that were up- and down-regulated either in Δ*bek1* or Δ*myt2*, which may be responsible for the observed difference in perithecium size (Fig. 5C). Indeed, *PKS13* (FGRRES_15980)—involved in zearalenone biosynthesis—was down-regulated in Δ*myt2*, consistent with the previous observation that Δ*myt2* produced a reduced amount of zearalenone (8).

There were noticeable phenotypic similarities between Δ*rel3* and Δ*vad3*. However, the DE genes identified in each knockout strain did not overlap, suggesting different roles in spore release (Fig. 5D). The only gene found to be differentially expressed in both was FGRRES_08011, encoding a CAZyme. To pinpoint genes implicated in spore release mechanisms, we specifically targeted a handful of DE genes that exhibited complete loss of expression or negligible expression in either Δ*rel3* or Δ*vad3* or both, and which were induced during later stages of perithecium development (Table S3). These genes encoded either hypothetical proteins or CAZymes. None of the knockouts lacking these DE genes exhibited detectable phenotypic changes.

### Co-expression and motif enrichment analyses

**Fig 6:**
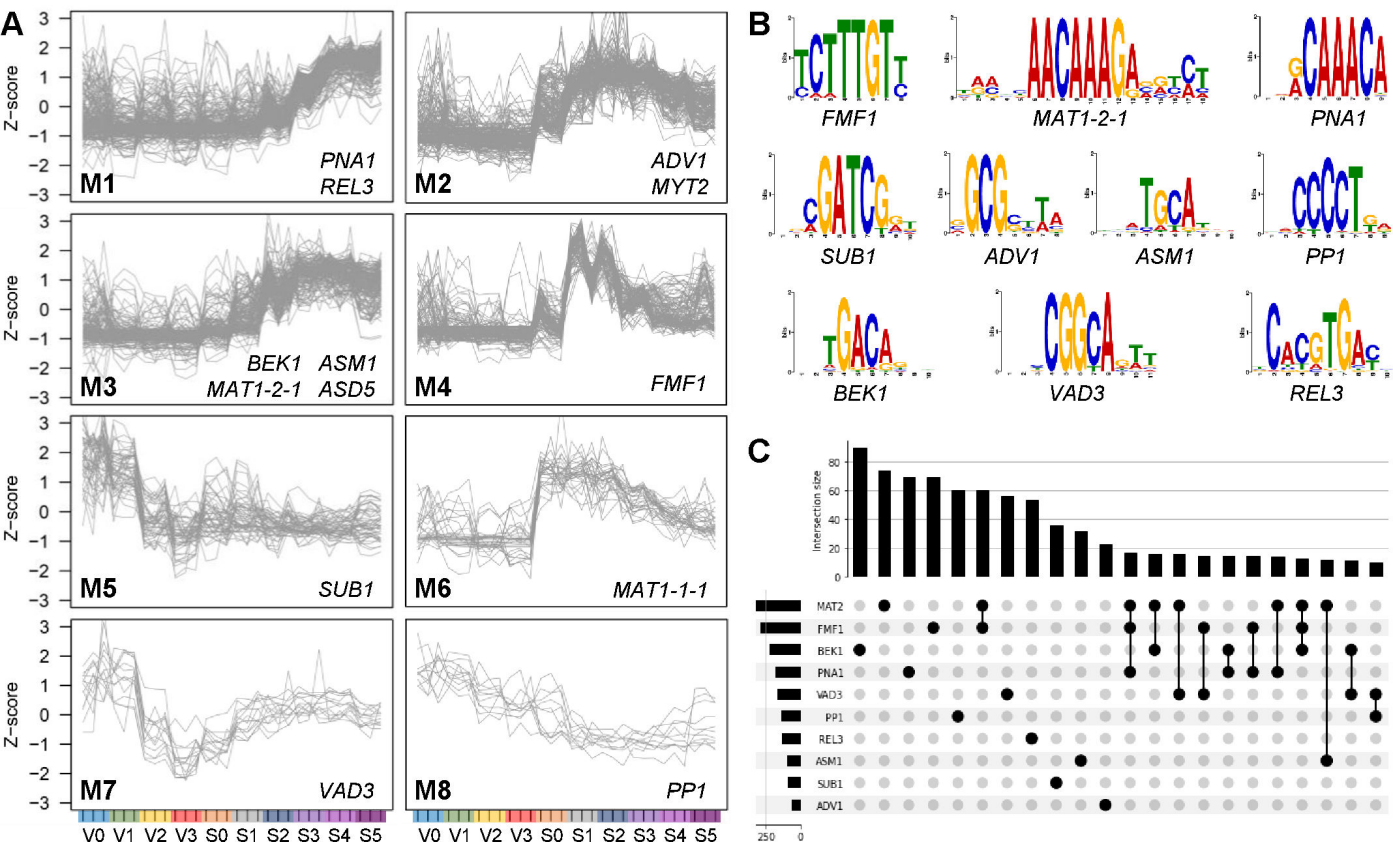
Co-expression network analysis during sexual reproduction. (**A**) Co-expressed modules of sexual stage-induced genes throughout the life cycle of *F. graminearum*. Vegetative stage 0 (**V0**): conidia harvested from carboxymethyl cellulose (CMC) medium (conidia stage); V1: 15 min after incubation on Bird medium (germination stage); V2: after 3-h incubation (polar growth stage); V3: after 11-h incubation (hyphal branching stage); sexual stage 0 (**S0**): after 2-h sexual induction on Carrot agar medium; S1: after 24-h sexual induction (induction stage); S2: after 48-h sexual induction (protoperithecium initials stage); S3: after 72-h sexual induction (paraphysis stage); S4: after 96-h sexual induction (ascus stage); S5: after 144-h sexual induction (ascospore stage). Trend plots of *Z*-score normalized expression values for genes in the modules (M1–M8) including the selected TFs as members are presented. (**B**) DNA-binding sequence motifs of the selected TFs inferred from other fungi. (**C**) An UpSet plot of DNA-binding sequence motifs identified in the promoters of sexual stage-induced genes. The graph on the lower left side displays the total number of genes (x-axis) that harbor the indicated TF’s DNA-binding sequence motifs (y-axis). The bar graph above exhibits the intersection of sets of motifs (y-axis).

To identify genes that were co-expressed with the 13 TFs, we focused on those genes induced during perithecium development. A total of 1,335 DE genes were identified from the wild-type transcriptome data set in the comparisons between successive sexual development stages (e.g., stage 1 vs stage 2). We also incorporated 281 genes that had been previously recognized for their importance in sexual reproduction into the co-expression network analysis. We identified 18 co-expression modules during the entire life cycles of the fungus, spanning vegetative growth (V0–V3) and sexual reproduction (S0–S5). Eight of these co-expression modules were associated with the 13 TFs (Fig. 6A). The other modules largely consist of genes that were expressed during vegetative stages (Fig. S3A and B).

The fact that closely related TFs within the same family almost always have very similar DNA sequence preferences for the recognition of targets enables accurate inference of TF-binding sites in eukaryotes (44). We extracted TF-binding sites, typically represented as “motifs,” for the selected TFs from the Catalog of Inferred Sequence Binding Preferences (CIS-BP) database, except for *ASD5*, *MAT1-1-1*, and *MYT2*, for whom motifs were absent (44) (Fig. 6B; Table S4). Using these motifs, we conducted enrichment analyses to explore the prevalent motif(s) in each co-expression module. Module 1, which includes *PNA1* and *REL3*, exhibited a significant enrichment of the consensus binding site for *PNA1*, whereas enrichment for the *REL3*-binding site was not evident (Table 3). Module 4, featuring *FMF1*, was enriched with the binding sites for *FMF1* and *MAT1-2-1* (Table 3). Also, *FMF1*-binding sites were prevalent in the promoter regions of genes belonging to modules 2 and 3 whose members exhibited sexual stage-specific expression patterns. The distribution of binding sites for the selected TFs suggested mostly exclusive presence in the promoters of sexually induced genes (Fig. 6C). Substantial numbers of genes contained the binding sites of both *FMF1* and *MAT1-2-1* in their promoter regions. This overlap is likely due to the palindromic nature of the binding sites for these two HMG-box-containing TFs. It was notable that *FMF1*-, *MAT1-2-1*-, and *PNA1*-binding sites sometimes overlapped each other and other TF-binding sites (Fig. 6C), suggesting their cooperative roles with other TFs in post-mating response.

**TABLE 3:** Analysis of motif enrichment for the selected transcription factors

Module or category (no. of genes)	Motif (ID)	Consensus seq.	Sites	Adjusted *P*-value*^a^*
Module				
M1 (279)	PNA1 (M01993_2.00)	NRRCAAACAN	93	2.98E−05
M2 (192)	FMF1 (M02491_2.00)	TCTTTGTY	33	1.49E−02
M3 (183)	FMF1 (M02491_2.00)	TCTTTGTY	61	1.06E−03
	PNA1 (M01993_2.00)	NRRCAAACAN	57	2.52E−03
M4 (121)	FMF1 (M02491_2.00)	TCTTTGTY	108	8.50E−19
	MAT2 (Kim et al. 2015)	ATTGTTTAA	24	9.58E−09
	MAT1 (M11340_2.00)	TRMNCAACAAAGAVRYMT	71	4.44E−07
M9 (164)	REL3 (M01112_2.00)	YCACGTGAYH	51	1.43E−04
	PP1 (M01870_2.00)	DRCCCCTKRN	32	1.05E−03
DE genes				
Δ*pna1* at S2 (216)	PNA1 (M01993_2.00)	NRRCAAACAN	64	1.09E−03
	FMF1 (M02491_2.00)	TCTTTGTY	49	1.78E−03
Δ*asd5* at S2 (133)	PP1 (M01870_2.00)	DRCCCCTKRN	28	7.26E−04
Δ*sub6* at S0 (972)	FMF1 (M02491_2.00)	TCTTTGTY	314	1.22E−09
	PNA1 (M01993_2.00)	NRRCAAACAN	230	6.60E−03
	MAT1 (M11340_2.00)	TRMNCAACAAAGAVRYMT	284	2.17E−02
Δ*adv1* at S0 (271)	ADV1 (M02624_2.00)	VGCGSYTAN	53	2.37E−07
Δ*asm1* at S0 (939)	ASM1 (M01652_2.00)	DHMTGCAKDH	216	4.70E−08
Δ*vad3* at S5 (13)	VAD3 (M02614_2.00)	NNYCGGCAGTT	8	3.80E−03

^*a*^ Enriched motifs with adjusted *P*-value smaller than 5.00E−02 were reported.

To identify potential direct target genes for the selected TFs, we performed motif enrichment analyses on DE genes identified in TF-knockout experiments (Fig. 2 and 3). Significant enrichments of binding sites for *ADV1*, *ASM1*, *PNA1*, and *VAD3* motifs were observed in the DE genes in their respective knockouts (Table 3). Also, *FMF1*-binding sites were enriched in the promoters of DE genes identified in Δ*pna1*, which may account for many DE genes observed in both Δ*fmf1* and Δ*pna1* (Fig. 5B). Out of the 216 DE genes identified in Δ*pna1*, 64 (30%) and 49 (23%) genes harbored *PNA1*- and *FMF1*-binding sites, respectively, within their promoter regions. Among these DE genes, 16 genes possessed both binding sites. Most of these 16 genes are annotated as encoding hypothetical proteins and exhibited high induction of gene expression during sexual development (Fig. S3C). Intriguingly, many genes identified as DE in Δ*sub1* were found to contain *FMF1*, *MAT1-2-1*, and/or *PNA1*-binding motifs in the promoter regions (Table 3). The presence of these binding motifs implies that a substantial number of genes affected by deletion of *SUB1* at an initial stage of sexual reproduction may also be under the regulation of these other TFs that are crucial for perithecium development.

### Mapping transcription factor network during sexual reproduction

**Fig 7:**
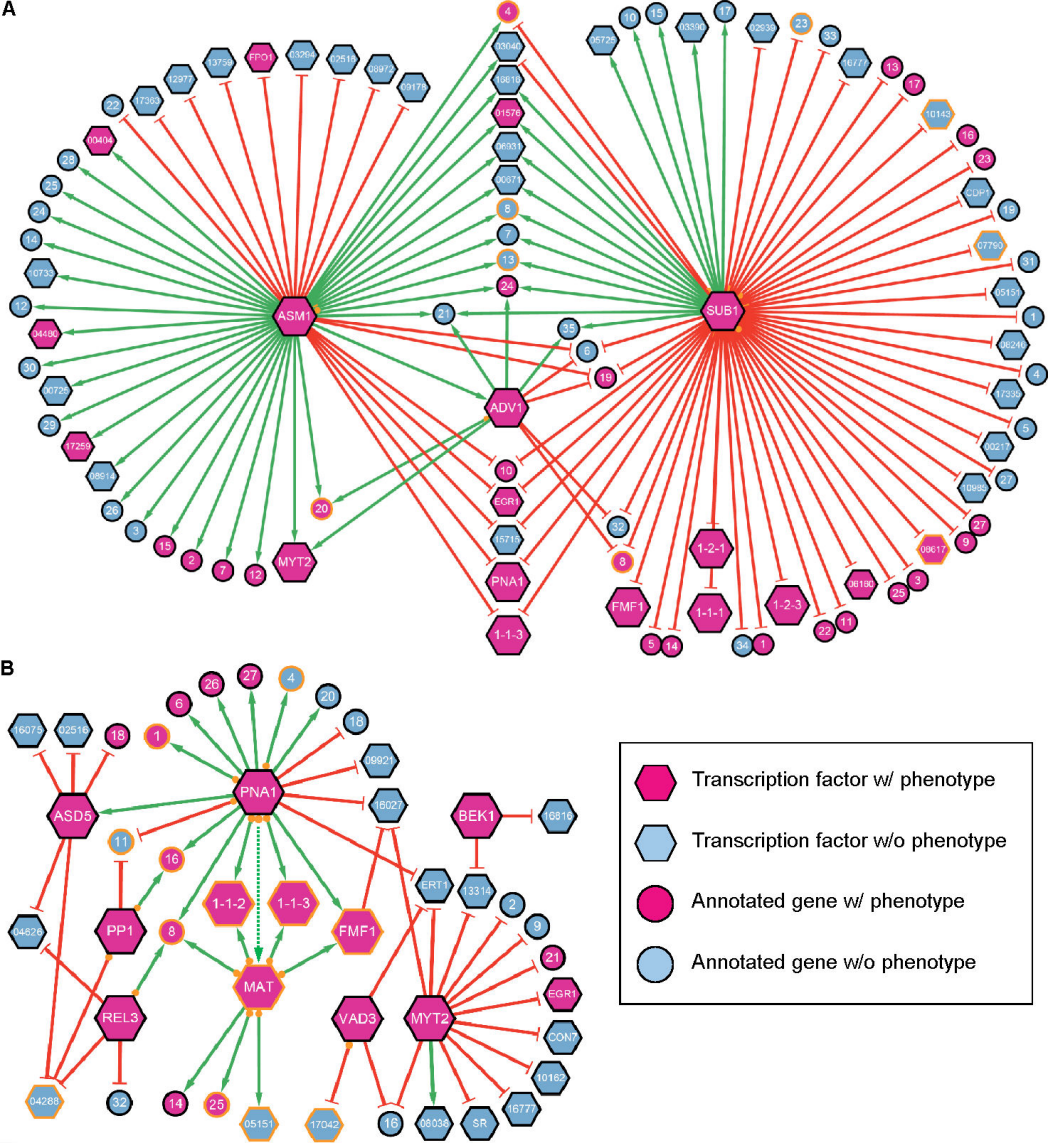
Gene regulatory networks between TFs and potential target genes during sexual reproduction. Edge-weighed spring-embedded layout of the networks is shown where nodes represent genes and edges represent regulatory interactions. Green arrows connect source nodes (the selected 13 TFs in this study) to DE genes down-regulated in the knockout of the corresponding TFs, while blunted red lines connect source nodes to DE genes up-regulated in the corresponding TF knockouts (inhibition). Lines connecting small orange circles on the side of source nodes to target nodes with orange borders indicate the presence of DNA-binding domains for the corresponding TFs (source nodes) in the promoter sequence of target nodes (potential direct target genes). These regulatory networks are sexual stage dependent, specific to (**A**) stage 0 and (**B**) stages 1–3. Detailed information on target nodes, such as gene ID, putative function, and knockout phenotypes, is provided in Table 4 for magenta nodes and in Table S5 for blue nodes. Note that a dashed green arrow connecting nodes for *PNA1* and *MAT1-1-1*/*MAT1-2-1* (labeled as *MAT*) is tentative, as *MAT1-1-1* was not a statistically significant DE gene in Δ*pna1*, despite being threefold down-regulated.

To chart transcriptional networks throughout sexual reproduction, we used annotated genes among the DE genes identified in TF knockouts, with a particular emphasis on genes crucial for sexual reproduction (Table 4; Table S5), as well as CAZymes and secondary metabolism-related genes (Fig. S4). Given the substantial number of DE genes identified in Δ*asm1* and Δ*sub1*, we opted to visualize transcriptional networks into two separate networks for 2 h after sexual induction (stage 0) and early stages of perithecium development (stages 1–3) to enhance visual clarity (Fig. 7).

**TABLE 4:** Information on magenta nodes in the regulatory networks in Fig. 7^*b*^

No.	Gene ID	Gene name	Up-regulated in*^a^*	Down-regulated in*^a^*	Putative function	Knockout phenotype	Reference
1	FGRRES_00348	*SMS2*	Δ*sub1*	**Δ*pna1***	Argonaute 2	Fewer asci; less ascospore discharge	(14)
2	FGRRES_00739	*NOX1*		Δ*asm1*	NADPH oxidase	No perithecia	(45)
3	FGRRES_02052		Δ*sub1*		Hypothetical protein	Faster cirrhi production	(16)
4	FGRRES_02102	*ASY1*	**Δ*sub1***	Δ*asm1*	Hypothetical protein	Asynchronous developing perithecia	(16)
5	FGRRES_02655	*PRE2*	Δ*sub1*		Pheromone receptor	Fewer perithecia; reduced female fertility	(46)
6	FGRRES_03673			Δ*pna1*	Carboxypeptidase	No asci	(47)
7	FGRRES_03916	*RDS1*		Δ*asm1*	Fibronectin-attachment protein	No perithecia	(14)
8	FGRRES_05061	*PPG1*	**Δ*sub1***; Δ*adv1*	Δ*pna1*; **ΔΔ*mat***; **Δ*rel3***	Pheromone precursor	Fewer perithecia; reduced male fertility	(46)
9	FGRRES_05239	*GIP1*	Δ*sub1*		G-protein coupled receptor-like	No perithecia	(47)
10	FGRRES_05325		Δ*sub1*; Δ*asm1*		Hypothetical protein	Small perithecia, no asci	(47)
11	FGRRES_05652	*ASY2*	Δ*sub1*		Hypothetical protein	Uneven perithecia growth	(16)
12	FGRRES_06774	*VOS1*		Δ*asm1*	Velvet complex component	N/D	(29)
13	FGRRES_06969	*FBP1*	Δ*sub1*		F-box protein	No asci	(48)
14	FGRRES_07270	*PRE1*	Δ*sub1*	ΔΔ*mat*	Pheromone receptor	Reduced fertility	(46)
15	FGRRES_07578			Δ*asm1*	3-Dehydroquinate synthetase	No asci	(14)
16	FGRRES_07869		Δ*sub1*	Δ*pna1*; **Δ*pp1***	Short chain dehydrogenase	Fewer perithecia, no asci	(47)
17	FGRRES_08576	*REC8*	Δ*sub1*		Meiotic cohesion	Abnormal ascospores	(26)
18	FGRRES_08695	*PLS1*	Δ*asd5*		Hypothetical protein	More perithecia	(16)
19	FGRRES_09896	*ICL1*	Δ*sub1*; Δ*asm1*; Δ*adv1*		Isocitrate lyase 1	No perithecia	(16)
20	FGRRES_10742			Δ*asm1*; **Δ*adv1***	Pheromone-regulated membrane protein	Small perithecia, no asci	(14)
21	FGRRES_12164	*FGP1*	Δ*myt2*		Wor1-like protein	Less ascospore	(49)
22	FGRRES_13162	*PDV2*	Δ*sub1*		Hypothetical protein	Small perithecia, no asci	(16)
23	FGRRES_15858		Δ*sub1*		Hypothetical protein	Fewer perithecia	(14)
24	FGRRES_15909	*EED*		Δ*sub1*; Δ*asm1*; Δ*adv1*	Polycomb repressive complex 2	Female sterility	(50)
25	FGRRES_16465		Δ*sub1*	**ΔΔ*mat***	Hypothetical protein	No paraphyses senescence	(14)
26	FGRRES_17022	*GEA1*		Δ*pna1*	Hypothetical protein	Abnormal ascospore morphology	(51)
27	FGRRES_17056		Δ*sub1*	Δ*pna1*	Cytochrome P450 monooxygenase	Small perithecia, no asci	(14)

^*a*^ Knockout of a TF gene in bold indicates the presence of TF-binding motif in the target gene.

^*b*^ N/D, not determined.

The phenotypic analysis of TF knockouts revealed distinct roles of *ASM1* and *SUB1*. The knockout Δ*asm1* was devoid of perithecia, underscoring the essential role of *ASM1* for the initiation of sexual reproduction. In contrast, Δ*sub1* was a prolific producer of submerged protoperithecia, highlighting the inhibitory effect of *SUB1* on perithecium development (Fig. 2B). Transcriptomic data from our knockout studies supported their distinct roles at a transcriptional level. *ASM1* seemed to positively regulate several TFs, including *ADV1*, FGRRES_00404, FGRRES_04480, and FGRRES_17259, whose knockouts resulted in sterility (8, 14) (Fig. 7A). Although *ASM1*-binding sites were not detected in the promoter regions, the defective phenotype observed in Δ*asm1* can be attributed to the down-regulation of these TF genes essential for sexual reproduction. On the other hand, there were some TF genes up-regulated in Δ*asm1*, such as *FPO1*, whose knockout leads to abnormal increase in the number of perithecium (33). In addition, *MAT1-1-3* and *PNA1* were up-regulated in Δ*asm1*.

In contrast, the expression of many TF genes that play important roles in the protoperithecium development, including *FMF1*, *MAT1-1-1*, *MAT1-1-3*, *MAT1-2-1*, and *PNA1*, was up-regulated in Δ*sub1* (Fig. 7A). Among TF genes up-regulated in Δ*sub1*, FGRRES_07790, FGRRES_08617, and FGRRES_10143 exhibited *SUB1*-binding sites in their promoter regions (Table S6). Notably, the promoter region of *ASY1*, a gene linked to uneven production of mature perithecia when knocked out (16), contained a *SUB1*-binding motif (Table S6). The expression of *ASY1* was up-regulated in Δ*sub1* and down-regulated in Δ*asm1*, emphasizing their opposing roles in sexual reproduction (Fig. 7A; Table 4). Nonetheless, there were also many genes commonly up- and down-regulated in both Δ*asm1* and Δ*sub1*, suggesting intricate crosstalk between *ASM1* and *SUB1* regulatory networks. A pheromone-regulated membrane protein gene (FGRRES_10742) exhibiting aborted perithecium development upon knockout (14) was down-regulated in Δ*adv1,* and its promoter region contained an *ADV1*-binding site (Table S6). This gene was also down-regulated in Δ*asm1*, which was likely mediated by down-regulation of *ADV1* in Δ*asm1* (Fig. 7A). This transcriptional cascade, involving *ADV1* and *ASM1*, was also observed in the regulation of *MYT2* that was down-regulated in both Δ*adv1* and Δ*asm1*.

The phenotypic analysis of TF knockouts crucial for sexual reproduction revealed that while Δ*fmf1*, ΔΔ*mat*, Δ*pna1*, and Δ*sub1* were all arrested at the protoperithecium stage, Δ*pna1* produced much smaller protoperithecia compared to the others (Fig. 2B; Fig. S2A). Our DE analysis indicated down-regulation of *FMF1*, *MAT1-1-2*, and *MAT1-1-3* in Δ*pna1* (Table S2). In addition, although statistically non-significant, the expression levels of other *MAT* genes (such as *MAT1-1-1*, *MAT1-2-1*, and *MAT1-2-3*) were lower by more than twofold in Δ*pna1. PNA1*-binding sites were found in the promoter regions of *MAT1-1-1* and *MAT1-2-3*, as well as *MAT1-1-2* and *MAT1-1-3* that were differentially expressed in Δ*pna1* (Table S6), suggesting that *PNA1* acts as an upstream regulator for these *MAT* genes (Fig. 7B). Several genes, whose knockout abolished the formation of asci and ascopores, were down-regulated in Δ*pna1*, including *ASD5* (a C_2_H_2_-type TF), FGRRES_00348 (*SMS2*/*AGO2*, known as argonaute 2), FGRRES_03673 (encoding a carboxypeptidase), FGRRES_05061 (*PPG1*, a pheromone precursor gene), FGRRES_07869 (encoding a short chain dehydrogenase), FGRRES_17022 (*GEA1*), and FGRRES_17056 (encoding a P450 enzyme) (Fig. 7B; Table 4). Within these DE genes in Δ*pna1*, a *PNA*-binding site was discovered in the promoter region of *SMS2* (Table S6).

Two HMG-box domain TFs, namely *FMF1* and the ortholog of *PaHMG8* (FGRRES_05151), along with the divergently transcribing *MAT1-1-2* and *MAT1-1-3*, appeared to be direct downstream targets of *MAT1-2-1* (Fig. 7B). This transcriptional cascade was supported by their down-regulation in ΔΔ*mat* and the presence of *MAT1-2-1*-binding sites in their promoters (Table S6). The pheromone precursor *PPG1* appeared to be activated by *MAT1-2-1* and *REL3*, while being suppressed by *SUB1*, as suggested by the presence of binding sites in the promoters and expression levels in their respective knockouts (Fig. 7). Although no TF-binding motifs in its promoter were detected, the pheromone receptor *PRE1* exhibited a similar expression pattern in ΔΔ*mat* and Δ*sub1*. Notably, an *MAT1-2-1*-binding site was identified in the promoter region of FGRRES_16465, a gene that plays an important role in paraphyses senescence and asci formation (14) (Table S6). FGRRES_16465 was down-regulated in ΔΔ*mat*, whereas it was up-regulated in Δ*sub1*. This contrast is consistent with the notion that *SUB1* antagonizes TFs crucial for sexual reproduction.

## DISCUSSION

We dissected global gene regulatory networks that function during sexual reproduction in *F. graminearum* by inferring Bayesian regulatory networks using transcriptomics data from key stages of sexual development, predicting stage-specific impacts of genes on *in silico* knockout networks, and sequentially by conducting RNA-seq analyses for knockout mutants for 13 TFs that play indispensable roles during different stages of perithecium development. This combination of network modeling, *in silico* manipulation, and gene knockout phenotyping provided a thorough assessment of gene regulatory mechanisms behind the complexity of sexual development in this important fungal pathogen. With limited prior knowledge, the Bayesian network predicted regulatory hubs with selected genes, providing powerful basis for *in silico* manipulation guided by the P0PCorNS criterion. The P0PCorNS criterion estimated stage-specific impacts of genes within the overall stage-specific knockout network space. Therefore, it served twofold purposes here: (i) it provided a means for inference of the robustness of network association within the regulatory hub in a predicted ensemble of networks; (ii) it provided guidance and prioritization of laboratory knockout perturbation via estimation of the impact on network knowledge likely to be conferred by stage-specific assessment of gene expression in knockout strains of genes. This approach provides powerful experimental design in investigations of moderate-sized networks with multiple-point expression data even for sets of genes that are not already well annotated with defined processes or that feature poorly understood gene-developmental complexities. It is especially appropriate when previous gene manipulations led to ambiguous conclusions or produced no obvious phenotypes. In this study, with gene-expression data at six developmental stages during perithecium formation, the predicted TF regulatory network suggested those TFs are multi-edged with a hub of *PNA1*, *ADV1*, and *ASM1* being more central. Interestingly, these three genes exhibited phenotypes in early sexual development, and they likely function as upstream regulators across the whole process. Additionally, more densely sampled gene-expression data, at later developmental stages, will provide additional power to biological inference guided by P0PCorNS. Moreover, inclusion of additional genes in the BN analysis will provide better prediction of how gene-expression dynamics affect the robustness for the predicted networks based on the P0PCorNS criterion.

TF-binding sequence preferences can accurately be inferred from previously known binding motifs in related species, by comparison of the overall sequence identity of DNA-binding domains within the same TF family (44, 52). Although experimental evidence, such as ChIP-seq-binding peak sequences or *in vitro* SELEX data, was lacking, we investigated potential direct targets of the selected TFs in *F. graminearum* through binding sites previously determined in orthologous genes for these TFs in *N. crassa*, *A. nidulans*, and fission yeast (44, 53–55). Importantly, we identified *PNA1* as a key regulator for triggering sexual reproduction, and revealed its central roles not only for the activation of *MAT* genes, but also for activating genes crucial for normal ascospore development, such as *AGO2*, the core component of the RNAi machinery (56, 57), and *GEA1*, whose knockout produced abnormal ascospores in morphology (51).

**Fig 8:**
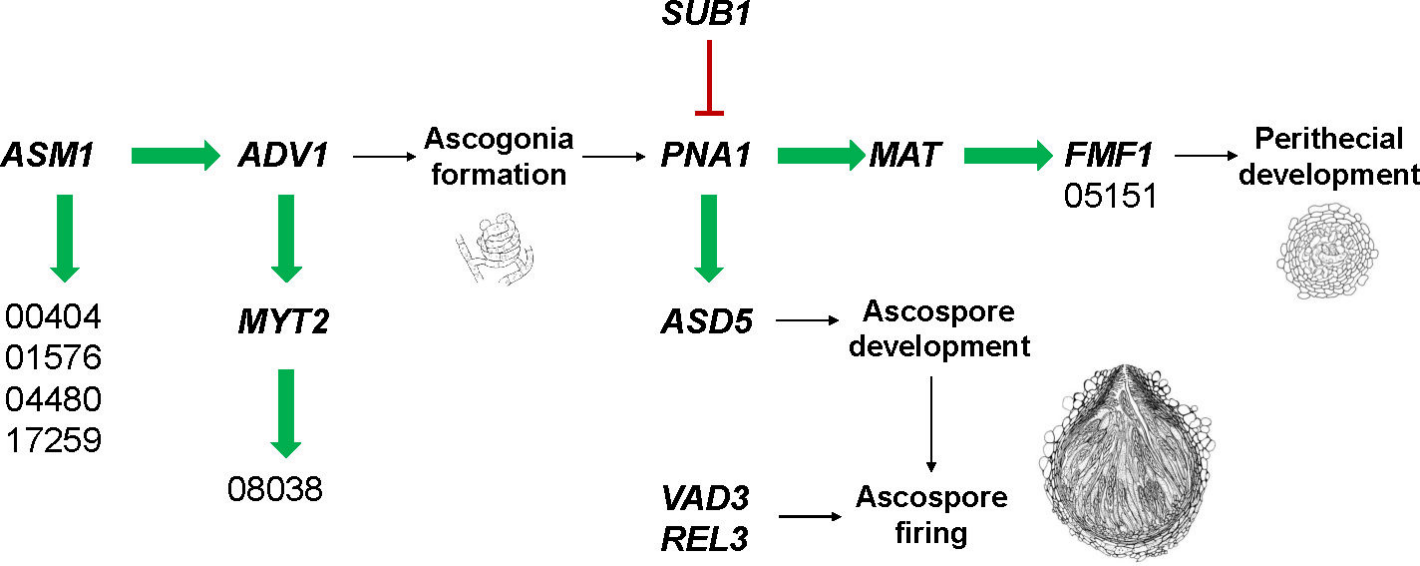
A proposed transcription factor network during sexual reproduction. A map of the regulatory interactions among TFs examined in this study, based on the RNA-seq data in the *F. graminearum* PH-1 strains deleted for each TF. Green arrows indicate positive regulation, while a blunted red line indicates negative regulation. Black arrows indicate the developmental progress during sexual reproduction. Five-digit numbers are gene ID, omitting the FGRRES prefix.

*PNA1* (FGRRES_15782, old gene ID: FGSG_11826) was previously identified as a TF that acts downstream of *MAT1-2-1* and *FMF1* (14). However, in our knockout transcriptome data, there was no change in the expression level of *PNA1* in Δ*fmf1*. Indeed, the expression level of *FMF1* significantly decreased in Δ*pna1* and ΔΔ*mat*. Interestingly, *MAT1-2-1*-binding sites were found in HMG-box family TFs, such as *FMF1*, *MAT1-1-3*, and FGRRES_05151, all of which were down-regulated in ΔΔ*mat*. The presence of *PNA1*-binding sites in the promoter region of *MAT* genes, such as *MAT1-1-1*, *MAT1-1-2*, *MAT1-1-3*, and *MAT1-2-3*, coupled with the discovery of *MAT1-2-1*-binding sites in the promoters of HMG-box family TFs, including *FMF1*, suggested a cascading sequence of TFs in the order of *PNA1*, *MAT1-1-1* (and *MAT1-2-1*), and *FMF1* (and FGRRES_05151) (Fig. 8). Our transcriptome data covering the life cycle of *F. graminearum* support this model, as *MAT1-1-1* and *MAT1-2-1* were already expressed at stage 0, reaching the peak at stage 1, while the expression of *FMF1* and FGRRES_05151 was initiated at stage 1. In *P. anserina*, *PaHMG5* (the ortholog of *FMF1*) and *PaHMG8* (the ortholog of FGRRES_05151) were identified as upstream regulators of *MAT1-1-1* and *MAT1-2-1* in the transcription factor network (15). The deletion of *PaHMG8* caused sterility in *P. anserina*. However, a knockout lacking FGRRES_05151 did not show any noticeable phenotypic change in *F. graminearum* (8). These conflicting observations suggest that the transcriptional programs governed by TFs may have undergone rewiring in related species within the Sordariomycetes, as has been concluded in a previous analysis (16).

*PNA1* encodes a forkhead-box DNA-binding domain, also known as a winged helix domain. Forkhead-box TFs play important roles in diverse developmental processes in eukaryotes (58). Many members of the forkhead-box TF family have been identified as “pioneer” factors capable of binding condensed chromatins and actively opening up local chromatin structures, thereby rendering them accessible for other TFs (59). Our data suggest that *PNA1* acts as a pioneer factor in initiating sexual reproduction. Deletion of *PNA1* resulted in nearly complete shutdown of the expression of the divergently transcribed *MAT1-1-2* and *MAT1-1-3* (which share a promoter sequence). However, expression of *MAT1-1-1* in Δ*pna1* decreased only to one-third of that in the wild-type, indicating that there may be regulatory factors other than *PNA1* for the control of *MAT1-1-1* gene expression. An informative next step would be to elucidate the upstream regulator or regulators of *PNA1*, and the signal transduction pathways independent of *PNA1* that coordinate transcriptional programs during sexual reproduction.

Although deletion of *SUB1* resulted in the up-regulation of *FMF1*, *PNA1*, *MAT* genes and FGRRES_05151, *SUB1*-binding sites were not discovered in the promoter regions of the TFs involved in the early stages of sexual development. Based on the TF regulatory cascade, *SUB1* directly or indirectly suppressed the expression levels of the pioneer TF *PNA1*, which in turn may affect the expression levels of *MAT* genes and *FMF1* in Δ*sub1* (Fig. 8). Down-regulation of *SUB1* appears to have caused derepression of several genes essential for perithecium development, such as *AGO2*, *PPG1*, *PRE1*, a short chain dehydrogenase (FGRRES_07869), and a P450 enzyme (FGRRES_17056), which may have led to ectopic production of protoperithecia in Δsub1. Supplementation of fatty acids in the form of Tween 60 led to an excessive formation of protoperithecium in Δsub1. The dramatic increase in expression levels of two pheromone receptor genes (*PRE1* and *PRE2*) and a pheromone precursor gene (*PPG1*) in Δ*sub1* (cf. no change in the expression of *PPG2*) may account for the ectopic formation of ascogonia and subsequent production of a number of aborted protoperithecia. The pheromone receptor and precursor genes are dispensable for perithecium development in *F. graminearum* (46). Nonetheless, the proper functioning of pheromone-related genes should increase the chance of fertilization. Interestingly, the expression of *PPG1* was sexual stage-specific, while *PPG2* was highly induced during conidial germination. In line with this observation, we identified the binding sites for *MAT1-2-1*, *REL3*, and *SUB1* within the promoter region of *PPG1*. The presence of these binding sites indicates that the expression of *PPG1* is intricately regulated by the interplay of multiple TFs during sexual development. Among the DE genes up-regulated in Δ*sub1*, we identified *SUB1*-binding motifs in the promoters of *ASY1* and a C_2_H_2_-type TF gene (FGRRES_08617) that caused reduced numbers of mature perithecia when knocked out (8, 16). *GIP1*, which encodes a non-pheromone G protein-coupled receptor, exhibited high up-regulation in Δ*sub1* (47). *GIA1*, a paralog of *GIP1* recently reported to be crucial for ascospore formation (60), was also up-regulated in Δsub1. These findings collectively indicate that SUB1 functions as a negative regulator for sexual reproduction.

The knockout strain Δ*asm1* was arrested at the vegetative stage, and the Δ*sub1* ectopically produced aborted protoperithecia. Accordingly, genome-wide gene expression of both Δ*asm1* and Δ*sub1* substantially deviated from that of the wild-type during sexual reproduction. Previously, gene regulatory networks in *F. graminearum* were shown to be compartmentalized; one gene module enriched with protein synthesis is induced during vegetative growth (25). Our functional enrichment analyses of DE genes suggested that *ASM1* and/or *SUB1* may regulate a gene module involved in translation and proteolysis. Interestingly, several TFs, including *ADV1* essential for perithecium development, were down-regulated in Δ*asm1*, which accounted for its pleiotropic phenotype. *MYT2* was the sole TF down-regulated in Δ*adv1*, suggesting a TF cascade: *ASM1*, *ADV1*, and *MYT2* (Fig. 8). The Δ*adv1* strain in *F. graminearum* did not produce protoperithecia, while the Δ*pro1* strain in *S. macrospora* produced protoperithecia, indicating that homologous TFs can play different roles in related species.

For TFs crucial to perithecium development, including *ASD5*, *FMF1*, *MAT* genes, and PNA1, there were no statistically significant functional enrichments in the knockout transcriptome data. This lack of determination of functional enrichment can be at least partially attributed to the fact that more than half of the DE genes encoded hypothetical proteins lacking functional annotations. This high degree of poorly understood genes indicates the potential for substantial increase in future understanding of the genetic basis of *F. graminearum* sexual development. The roles of these hypothetical genes remain unknown, but many of the hypothetical genes have been shown to be crucial for sexual reproduction in *F. graminearum* (14, 16, 26, 61, 62).

In our functional characterization of several DE genes identified in Δ*rel3* and Δ*vad3*, we found that most of them also encoded hypothetical proteins. Although we were unable to pinpoint genes directly responsible for spore firing, knockout experiments targeting a DE gene observed in Δ*vad3* (FGRRES_08203, encoding a protein domain related to choline dehydrogenase) resulted in the production of smaller perithecia. Notably, this gene harbors two *VAD3*-binding motifs within its promoter region, suggesting FGRRES_08203 as a probable direct target of VAD3. Previously, we have reported that calcium ion channels encoded by *CCH1* (FGRRES_07418) and *MID1* (FGRRES_01364) are important for forcible spore discharge in *F. graminearum* (63, 64). The fact that the expression levels of these two calcium ion channels were not altered by deletion of either *REL3* or *VAD3* indicates that the observed phenotypic defects are independent of the calcium channels. In our functional enrichment analysis, the GO category for monoatomic ion transport (GO: 0006811) was enriched in Δ*rel3*. Interestingly, three genes within this category, each encoding a ferric/cupric reductase transmembrane component (FGRRES_04780, FGRRES_10655, and FGRRES_17471), were highly up-regulated in Δ*rel3*. The expression profiles of these three genes exhibited similarity, showing induction during perithecium development and a rapid decline at stage 5, coinciding with active spore discharge (Fig. S3D). This stage-specific expression suggests that the absence of spore firing observed in Δ*rel3* may be partially attributed to ectopic expression of the metalloreductase, though its functional significance in sexual reproduction is yet to be demonstrated.

Conservation analysis of TF families across Sordariomycetes species revealed the presence of orthologs for most TF families crucial for sexual reproduction, underscoring their evolutionary significance. However, *Phaeoacremonium minimum* possessed only 71 TFs with phenotypes observed during sexual development, and most TFs could not be identified as orthologous to genes in other species. Although some species in the genus *Phaeoacremonium* have been previously connected to the teleomorph *Togninia*, many species, including *P. minimum*, have been described as hyphomycetes. The diminished number of TFs essential to sexual development in *P. minimum* may be related to its inability to form sexual stage or as-yet-unknown teleomorph.

A transcriptional cascade comprises a series of TFs that regulate one another, establishing a directed path within the gene regulatory network. In fungi, the cascade of gene expression during sexual reproduction initiates when cells perceive environmental cues for mating, leading to the activation of a set of genes, including *MAT* genes. In this study, we identified potential direct targets of selected TFs, with *PNA1* emerging as a key regulator triggering sexual reproduction, and *SUB1* acting as a negative regulator. Transcriptomic analyses unveiled intricate transcriptional cascades orchestrated by these TFs, shedding light on the complex regulatory interactions governing sexual reproduction. In future research, it will be of utmost interest to delve into the molecular activity of *PNA1* as a pioneer TF and verify its direct targets through ChIP-seq or DAP-seq analyses. This comprehensive study lays a solid foundation for further exploration of transcriptional cascades involving various TFs and better understanding of regulatory mechanisms during sexual reproduction in fungi.

## MATERIALS AND METHODS

### Ortholog identification for TFs

Among c. 600 TF-encoding genes in *F. graminearum*, we selected 93 TFs whose knockouts exhibited phenotypic defects during sexual reproduction in *F. graminearum* and related *Fusarium* species in the previous literatures (8, 14, 27–39). Protein sequences of 64 species in the Sordariomycetes and six species in the Leotiomycetes (as an outgroup) were downloaded from the NCBI Reference Sequence Database (www.ncbi.nlm.nih.gov/refseq/). To identify copy-number variation for the 93 TFs, orthology relationships of protein sequences in a total of 70 species in diverse taxonomic groups of fungi were inferred, using OrthoFinder (v.2.5.4) with default settings (65). The species tree of the 70 species was reconstructed using STRIDE (66), and the metadata for the NCBI reference genomes was tabulated (Table S1).

### Perithecium induction and RNA-seq analyses

For sexual stage induction, a 60 mm diameter plate with carrot agar medium (67) was inoculated by placing an agar block containing *F. graminearum* hyphae at its center, then maintained at room temperature exposed to constant light. After 6 days of incubation, the mycelia were gently scraped off the surface using a spatula. Subsequently, 0.9 mL of 2.5% Tween 60 (Sigma-Aldrich, St. Louis, MO, USA) was applied to the surface to facilitate perithecium formation. Perithecia in knockout mutants were observed for both size and quantity using stereomicroscopy at 7 days after sexual induction. Squash mounts of young developing perithecia in water were examined under a compound microscope to assess the morphology and maturity of asci and ascospores therein (Fig. S2). To obtain maximum information of transcriptional changes that can be observed in TF knockouts, total RNA samples were extracted from mycelia and perithecia at developmental stages prioritized by the JSD matrix. Strand-specific cDNA libraries were generated from poly-A captured RNAs, using the KAPA Stranded RNA-Seq Library Preparation Kit (Kapa Biosystems, Wilmington, MA), and sequenced on the Illumina HiSeq 2500 platform (Illumina Inc., San Diego, CA) at the Michigan State University’s Research Technology Support Facility (https://rtsf.natsci.msu.edu/genomics). Fifty base-pair single-end raw reads were further processed and filtered, using the TrimGalore (v.0.6.6) (https://www.bioinformatics.babraham.ac.uk/projects/trim_galore/). Filtered reads were mapped to the *F. graminearum* genome (NCBI accession: GCA_000240135.3), using the HISAT2 program (v.2.1.0). Mapped reads on exonic regions based on the Ensembl annotation version 32 were counted for each gene, using the htseq-count program (v.0.13.5). Gene-expression levels in counts-per-million value were computed and normalized by effective library size estimated by trimmed mean of M values, using the edgeR R package (v.3.26.8) (68). Only genes with counts-per-mapped reads (CPM) greater than or equal to one in at least three samples were kept for further analyses to exclude genes with low expression levels from the downstream analyses. Then, DE genes showing greater than fourfold difference—imposing a false discovery rate (FDR) no greater than 5%—were identified between the wild-type and a TF knockout, using the limma R package (v.3.40.6) (69).

### Constructing Bayesian networks and assessing impacts of TFs within the network

TF regulatory networks of sexual development in *F. graminearum* were inferred using gene-expression data collected from six stages across the process available from our previous study (41). Bayesian networks were modeled using the Bayesian Network Webserver (43). Global structure learning settings were retained at default settings. Each network depicted is the 50% majority consensus of the 100 highest-scoring models that retain edges exceeding a selection threshold of 0.5, performed without imposing any structural constraints. Due to the limited prior knowledge of TF roles and RNA-seq data points sampled during sexual development in this fungus, the robustness of the predicted regulator hubs in the TF regulatory networks was assessed via an *in silico* analysis. Namely, the Perturbation to 0 to Predict Correlated Network Solidity criterion, available at https://github.com/Townsend-Lab-Yale/P0PCorNS, was applied to the BN models with *in silico* knockout inputs.

The overall network topology, derived from wild-type data, is likely more responsive to alterations in central hub regulators compared to changes in peripheral regulators. First, the wild-type regulatory networks were generated using the Bayesian Network Webserver for biological network modeling (43). Default settings were used (the maximum number of parents for any node was set to be 4, number of networks to be included in model averaging was set to be 20, and the model-average edge-selection threshold was set to be 0.5), engaging structure learning without specifying network structural constraints. Bayesian networks were used to model stage-specific gene-expression manipulation *in silico*, predicting the effects of gene knockouts on the structure of the network. The wild-type expression levels for a given gene at a given stage were replaced with expression levels of 0 to infer a Bayesian network. For *in silico* prediction, wild-type expression of other genes were kept in the input, reflecting our lack of prior knowledge about the interaction dynamics among the TFs during the development of the complexity. The P0PCorNS criterion was used to prioritize gene knockout experiments based on the JSD. BN topologies were described with a matrix of posterior possibilities. All newly generated stage-specific knockout networks were compared to the wild-type network, using a JSD matrix of the dissimilarity between the two probability distributions that define the network topologies and edge-association strengths to be compared. JSD is commonly used to quantify deviation or divergence between network topologies (70, 71). Such divergence measurements enable *in silico* quantification of the overall knockout impacts that can be used to quantitatively assess and rank the robustness of the interactions among genes in a given network, to identify possible regulatory hubs and peripheral players, and thus to guide laboratory gene-manipulation experiments.

### Generation of TF-knockout mutants

To generate gene deletion mutants of the 13 conserved TF genes in *F. graminearum* PH-1 strain, we utilized a split marker approach (72). This method involved amplifying the left and right flanking regions of the target TF genes and combining them with a minimal gene cassette containing the hygromycin phosphotransferase (HPH) gene controlled by the trpC promoter from *A. nidulans*. The fusion PCR technique was employed as previously described (73). The primer sequences used in targeted gene deletion can be found in Table S7. In brief, we separately amplified the left and right flanking regions of the target genes using L5 and L3 primer pairs and R5 and R3 primer pairs, respectively. The L3 and R5 primers had 27 nucleotide (nt)-long overhang sequences complementary to the 5´ and 3´ ends of the minimal HPH cassette (1,376 bp). The HPH cassette in the pCB1004 plasmid (74) was amplified using HYG-F and HYG-R primers. The PCR amplicons were then merged through overlap extension, combining the left flanking region with the HPH cassette and the right flanking region with the HPH cassette. The split marker constructs were generated by amplifying the fused amplicons using nested primer pairs (N5 and HY-R primers for the left-half construct and YG-F and N3 primers for the right-half construct). Finally, we introduced the two split marker constructs into protoplasts through polyethylene glycol-mediated transformation (75). We usually obtained a handful of transformants from a single transformation event. Protoplasts are spread out in the selection media and hygromycin-resistant transformants are regenerated from the overlaid media, each forming colonies. Individual colonies are formed as a result of independent recombination events in different protoplasts. Several transformants were screened for replacement of the target gene with the HPH cassette through diagnostic PCR checks (Fig. S5). L5 and R3 primers, annealing to flanking sequences of the homologous recombination event, were used to confirm the homologous integration of the HPH cassette into the target gene locus. We observed at least two transformants to check the consistency of knockout phenotypes.

### Functional enrichment analysis

GO terms for 16,663 genes annotated in *F. graminearum* strain PH-1 were downloaded from the UniProt database (https://www.uniprot.org/) (accessed on 2 November 2023). Functional enrichment analysis for DE genes in TF knockouts were performed using the GOseq R package (v.1.36.0), excluding DE genes with no assigned GO term. To assess enrichment of GO terms, the Wallenius approximation (an extension of the hypergeometric distribution) and Benjamini-Hochberg method were used to calculate the FDR-corrected *P*-value.

### Cytoscape network visualization

To visualize DE genes observed in TF knockouts within a network space using the Cytoscape program (v.9.3.1), we selected genes that have been previously described in the literature, including genes with knockout phenotypes reported to be defective in sexual reproduction. Several genes were repeatedly detected as DE genes in different TF knockouts, such as FGRRES_02502 (*ERG3*, involved in ergosterol biosynthesis), FGRRES_16873 (*STC1*, encoding a terpene synthase), FGRRES_03687_M, FGRRES_03816_M, FGRRES_10224, and FGRRES_17390. As TFs regulate specific sets of genes, these genes may be involved in common downstream pathways at early stages of sexual development, and may not be targets of TFs examined in this study. Thus, we excluded these genes from the TF network analysis. For brevity, two networks were separately visualized: one connecting three TFs (*ASM1*, *ADV1*, and *SUB1*) with DE genes at stage 0, and the other connecting nine TFs (*ASD5*, *BEK1*, *FMF1*, *MAT*, *MYT2*, *PNA1*, *PP1*, *REL3,* and *VAD3*) with DE genes at stage 1 or stage 2. Visualization was achieved using the edge-weighted spring-embedded layout in the Cytoscape program (v.3.9.1).

### Gene co-expression network analysis

A non-redundant list of 1,335 sexually induced genes was derived from DE analyses, comparing two successive perithecium stages using the limma R package, as described previously (41). Additionally, 281 genes, serving as nodes in the Cytoscape networks, were included for the weighted gene correlation network analysis (WGCNA), bringing the total number of genes to 1,544. To identify gene sets co-expressed with 13 TFs during the developmental stages, the WGCNA R package (v.1.72-1) was used to cluster 1,544 genes by log_2_-transformed reads per kilobase of transcript per million mapped reads (RPKM) values calculated from 30 RNA-seq samples, each representing triplicate data for 10 successive developmental stages, as defined in the previous study (76). The “pickSoftThreshold” function was used to determine soft-thresholding power that measures the strength of correlation based on not just the direct correlation value of pairs of genes, but also the weighted correlations of all of their shared neighbors in the network space. The soft-thresholding power 12 was selected, which is the lowest power for which the scale-free topology model fit index reaches 0.9. Treecut value for cluster detection was set to 0.1 (corresponding to correlation of 0.9), resulting in 18 co-expression clusters (modules) with a minimum size set to include three genes.

### DNA sequence motif analyses

TF-binding sequences, typically represented as “motifs,” for the selected TFs were retrieved from the CIS-BP database (http://cisbp.ccbr.utoronto.ca/) (44); the motifs for the MAT1-2-1 had direct experimental evidence in *F. graminearum* (14). The motifs for the ADV1, FMF1, PNA1, PP1, SUB1, and VAD3 were confidently inferred from the motifs for the orthologous genes in *N. crassa*, the model organism for studying Sordariomycetes fungi (44, 53), while the motifs for ASM1, BEK1, and REL3 were inferred from *Nectria haematococca*, *Mus musculus*, and *A. nidulans* (44, 53, 55) (Table S4). Promoter sequences (500 bp upstream of the predicted ATG start codon) for annotated genes in the PH-1 strain were extracted, using a bash script available at a GitHub repository (https://github.com/RimGubaev/extract_promoters). For the motif enrichment analysis, we identified over-represented motifs from the promoter sequences of DE genes in TF knockouts or sets of genes in the WGCNA modules, using the AME program in the MEME suite (v.5.5.4; 77). For a control background, 1,000 randomly chosen promoter sequences were used to test the enrichment. To investigate occurrences of the motifs in the promoter sequences of genes listed in the co-expression modules and DE genes in TF knockouts, we used the FIMO program in the MEME suite (v.5.5.4; 78). Motif occurrences with a *P*-value less than 10^–4^ were reported.

## Data Availability

The RNA-seq data of TF knockouts generated in the present work have been deposited in the NCBI Sequence Read Archive (SRA) and are accessible through SRA accessions from SRX24161965 to SRX24162024, which belong to the BioProject accession PRJNA1096458. RNA-seq data for gene-expression levels throughout the life cycle of *F. graminearum* can be found in NCBI’s Gene Expression Omnibus GSE109088 for the conidial germination stages (G1–G3) and GSE109094 for the sexual development (S0–S5). RNA-seq data for the dormant conidia stage (G0) can be found in SRA accessions from SRX21157089 to SRX21157091, which belong to the BioProject PRJNA998561.

## SUPPLEMENTAL MATERIAL

Fig. S1 mbio.03030-24-s0001.pdf Gene expression levels of transcription factors important for sexual development.

Fig. S2 mbio.03030-24-s0002.pdf Perithecial formation.

Fig. S3 mbio.03030-24-s0003.pdf Co−expression modules.

Fig. S4 mbio.03030-24-s0004.pdf Gene regulatory networks between transcription factors and potential target genes related to CAZymes and secondary metabolite-related genes.

Fig. S5 mbio.03030-24-s0005.pdf Confirmation of the gene deletions by PCR.

Table S1 mbio.03030-24-s0006.xlsx Metadata for NCBI reference genomes and transcription factor genes.

Table S2 mbio.03030-24-s0007.xlsx Phenotypic analysis of genes putatively regulated by VAD3 and REL3.

Table S3 mbio.03030-24-s0008.xlsx Differentially expressed genes in transcription factor knockouts.

Supplemental tables mbio.03030-24-s0009.xlsx Tables S4 to S6.

Table S5 mbio.03030-24-s0010.xlsx Primers used in knockout generation.

ASM does not own the copyrights to Supplemental Material that may be linked to, or accessed through, an article. The authors have granted ASM a non-exclusive, world-wide license to publish the Supplemental Material files. Please contact the corresponding author directly for reuse.
