# Supplementary material for: Transcription factor-dependent regulatory networks of sexual reproduction in Fusarium graminearum
Source: mBio. 2024 Nov 26;16(1):e03030-24. doi: 10.1128/mbio.03030-24 (PMC11708053; doi:10.1128/mbio.03030-24)
Supplement: Fig. S1 — Gene expression levels of transcription factors important for sexual development. [file mbio.03030-24-s0001.pdf]

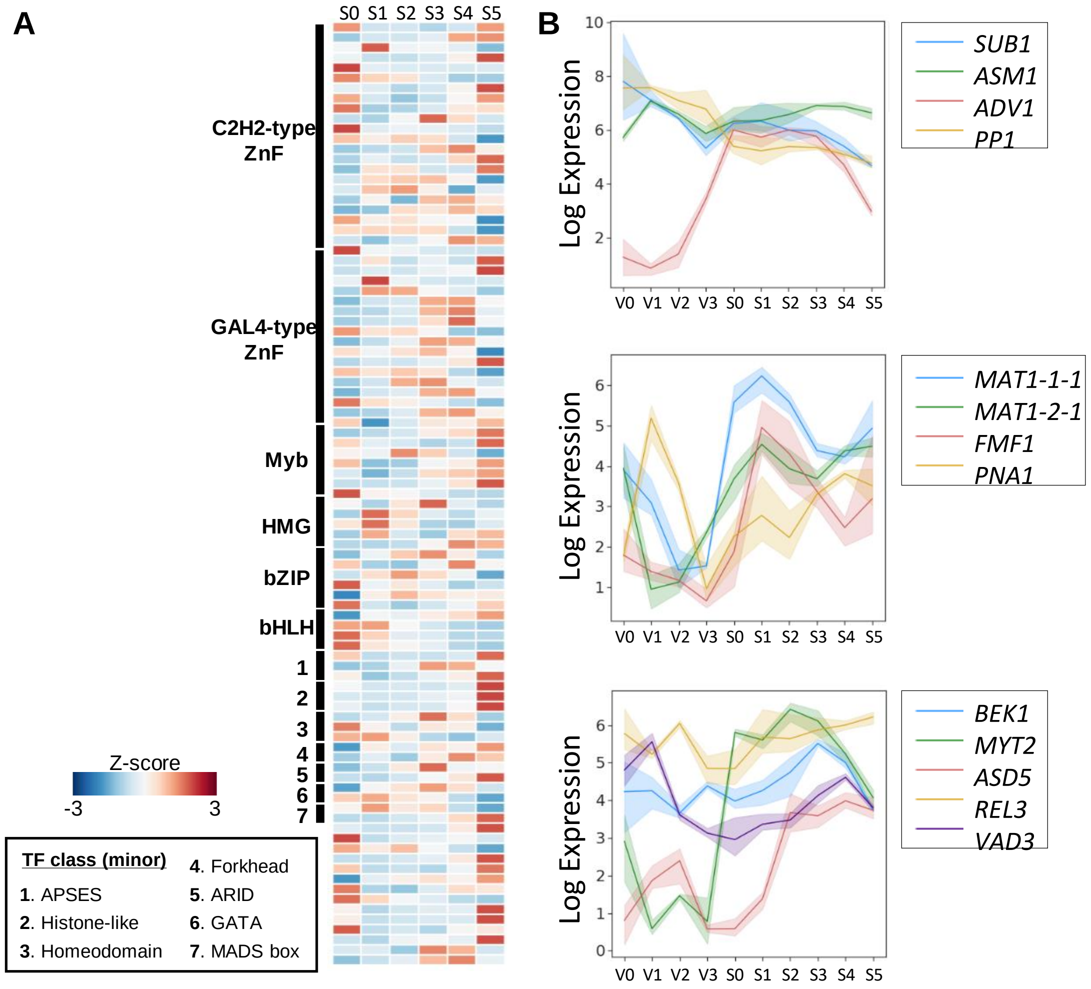

Supplementary Fig. S1. Gene expression levels of transcription factors (TFs) important for sexual development. (**A**) Heatmap of *Z*-score normalized reads per kilobase per million mapped reads (RPKM) values of 93 TFs across sexual development (S0−S5). (**B**) Gene expression profiles of selected 93 TFs in *Fusarium graminearum*. Averaged $\log_2$-transformed RPKM values were plotted. Bands surrounding the line plots indicate 95% confidence intervals of the mean of three replicate samples. The *x*-axis shows growth stages of *F. graminearum*: V0: Vegetative stage 0, conidia harvested from CMC medium (conidial stage); V1: after 15 min incubation on Bird medium (germination stage); V2: after 3 h incubation on Bird medium (polar growth stage); V3: after 11 h incubation (hyphal branching stage); Sexual stage 0 (S0): after 2 h sexual induction on Carrot agar medium; S1: after 24 h sexual induction (induction stage); S2: after 48 h sexual induction (protoperithecium initials stage); S3: after 72 h sexual induction (paraphysis stage); S4: after 96 h sexual induction (ascus stage); S5: after 144 h sexual induction (ascospore stage).
