# Supplementary material for: Transcription factor-dependent regulatory networks of sexual reproduction in Fusarium graminearum
Source: mBio. 2024 Nov 26;16(1):e03030-24. doi: 10.1128/mbio.03030-24 (PMC11708053; doi:10.1128/mbio.03030-24)
Supplement: Fig. S2 — Perithecial formation. [file mbio.03030-24-s0002.pdf]

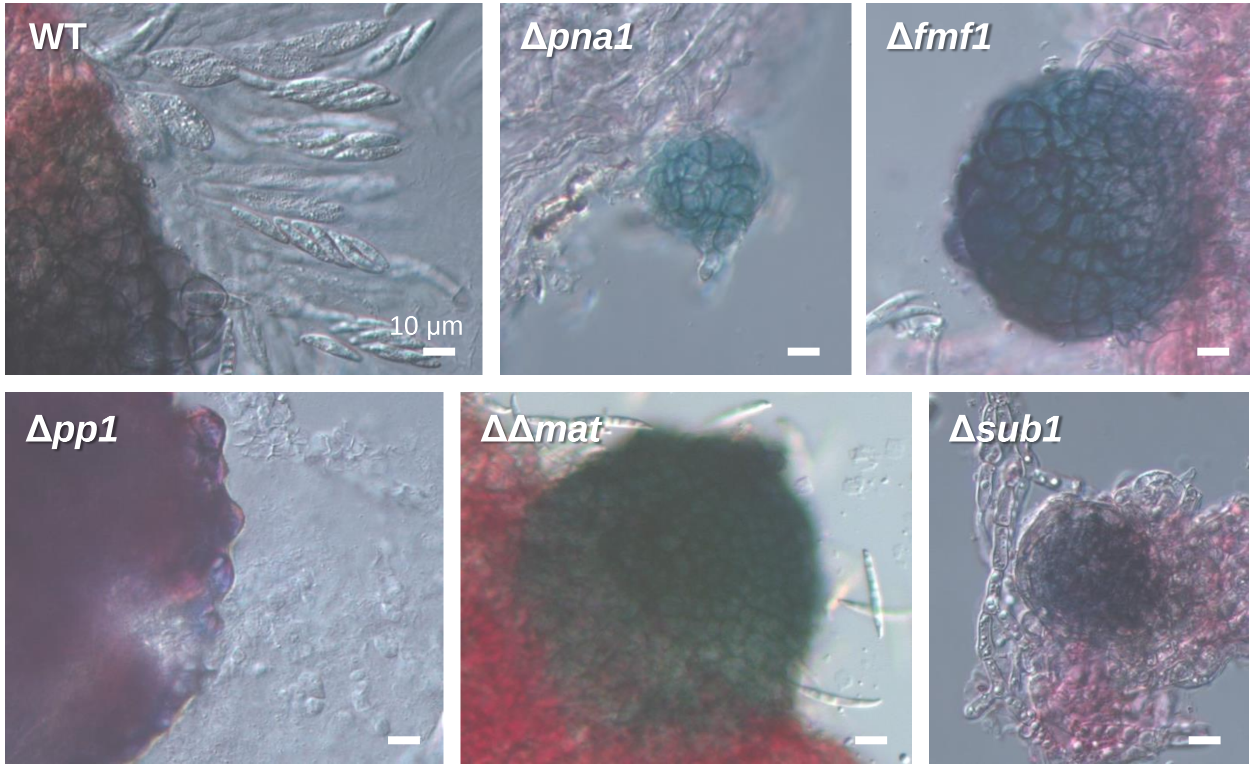

Supplementary Fig. S2A. Perithecia formation halted at a stage of protoperithecial development in transcription factor knockouts. Enlarged views of a squashed proto-perithecium devoid of cellular contents 7 days after sexual induction.

Tween-60 treatment No treatment

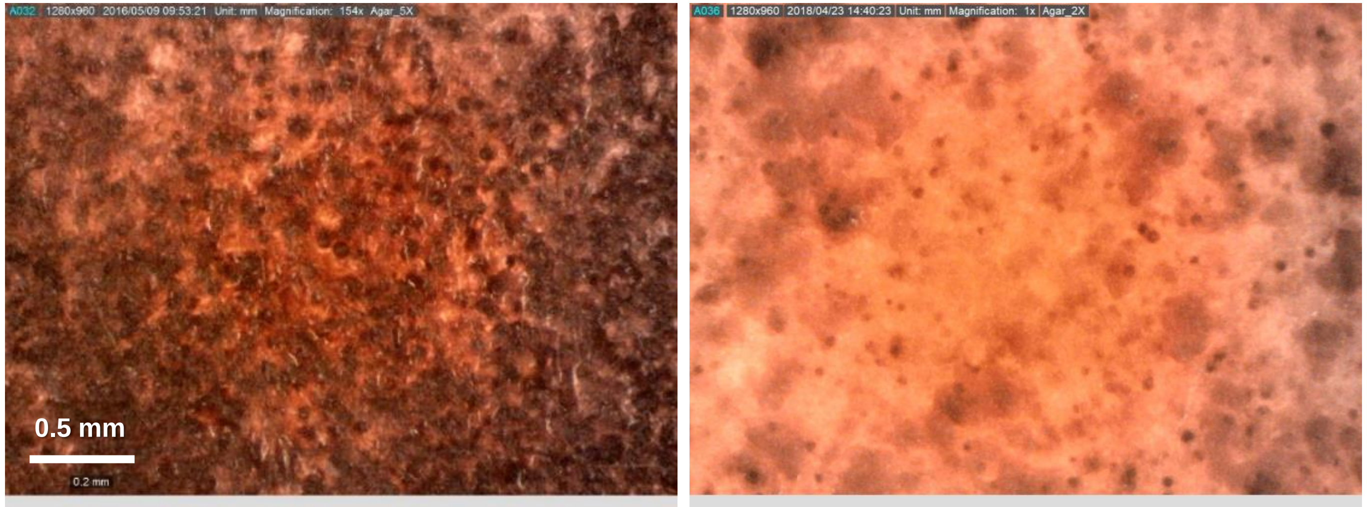

Supplementary Fig. S2B. Ectopic production of submerged protoperithecia in Δ*sub1*, especially when treated with 2.5% Tween-60 for sexual induction (left panel). No Tween-60 treatment (right panel). Photos were taken 7 days after sexual induction.

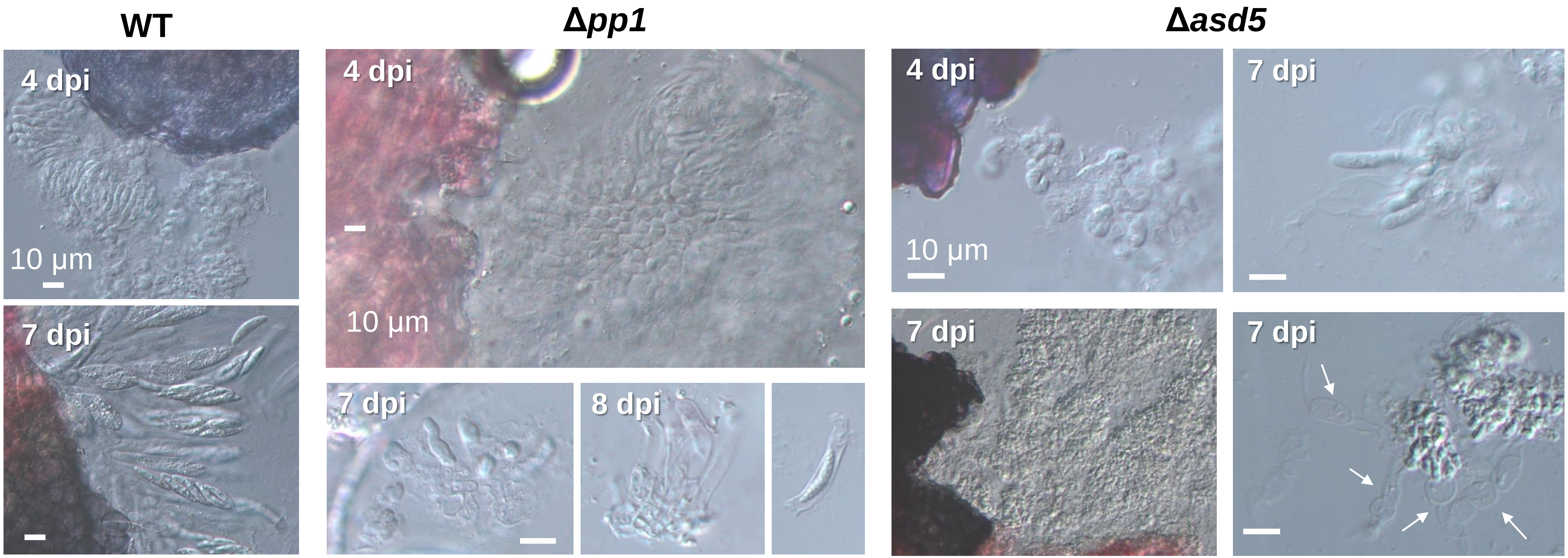

Supplementary Fig. S2C. Knockout phenotypes of *PP1* (left panels) and *ASD5* (right panels). At 4 days after sexual induction (dpi), formation of paraphyses in Δ*pp1* and croziers in Δ*asd5*. At 7dpi, formation of asci, although immature, is evident in Δ*pp1* and Δ*asd5*. However, no ascospore was found in squashed protoperithecia. At 8 dpi, although very rare, ascospores were found in Δ*pp1.* Arrows indicate senesced, swollen paraphyses.

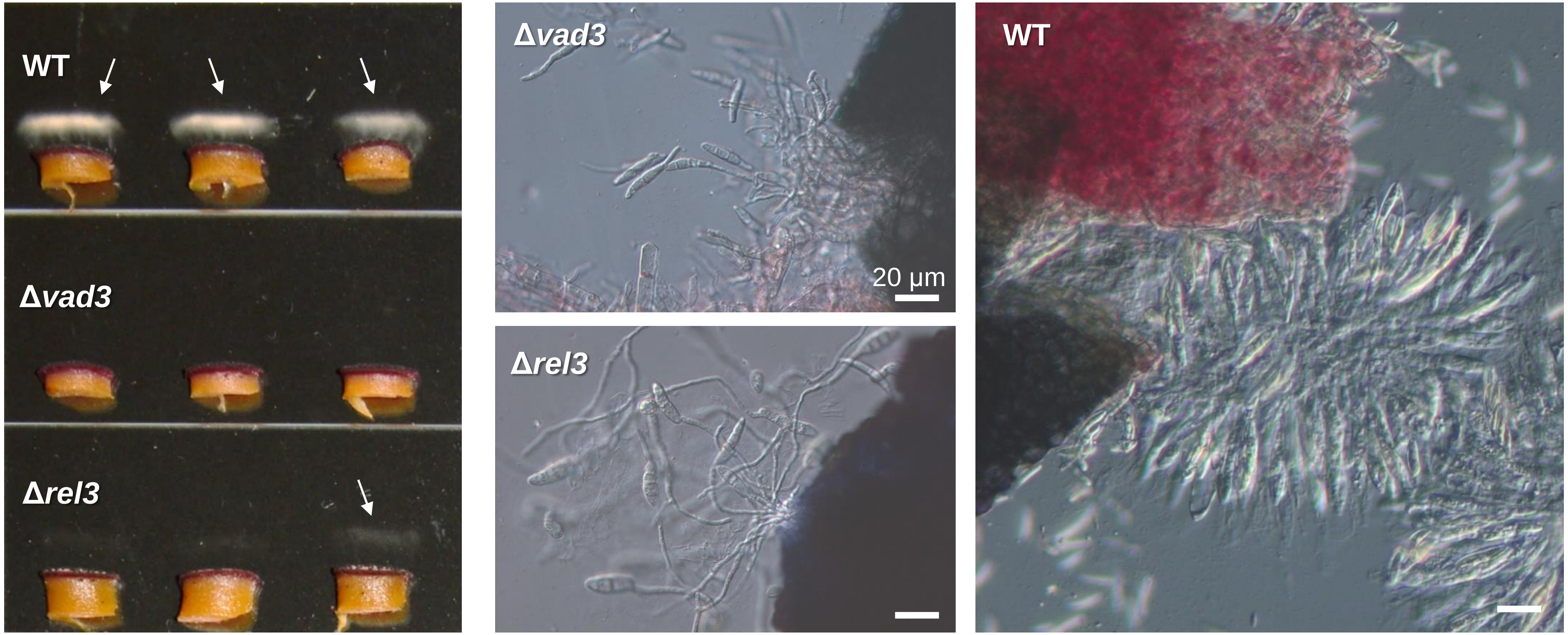

Supplementary Fig. S2D. Reduced ascospore discharge in knockout mutants of *VAD3* and *REL3* (left panels). Agar plugs supporting mature perithecia at 7 days after sexual induction (dpi) were oriented perpendicular to the glass slides so that spores fired and accumulated on the slides (arrows). Photos taken 3 days after the set up. Germinating ascospores inside the perithecium were observed in Δ*rel3* and Δ*vad3* at 10 dpi, causing a blockage of the ostiole (right panels).

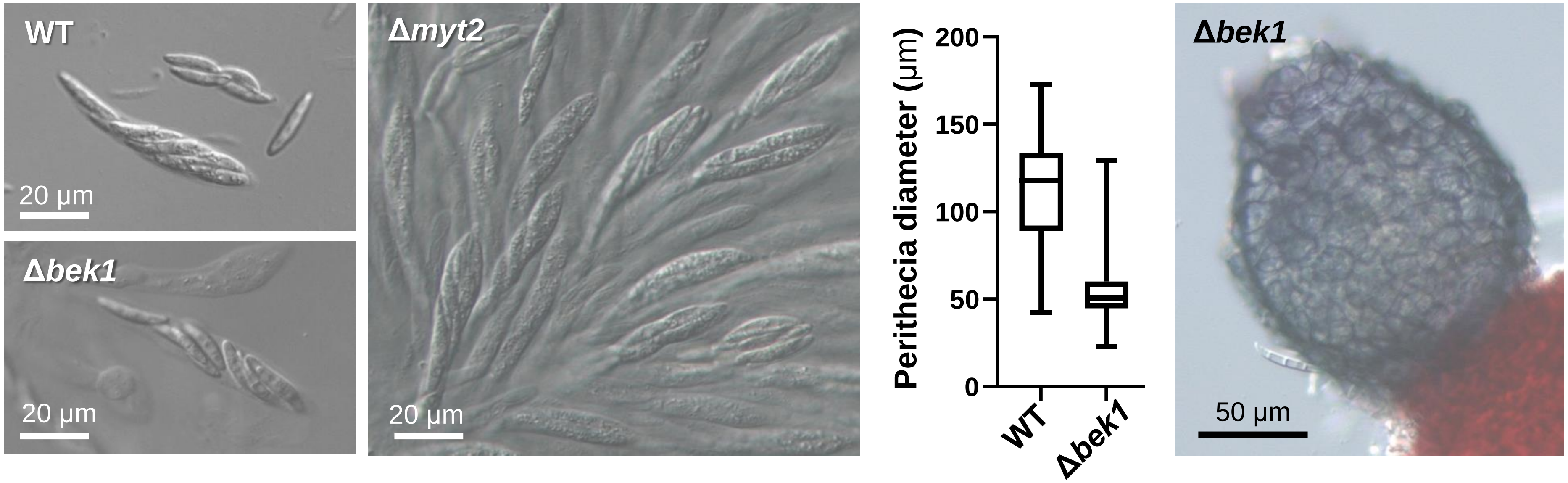

Supplementary Fig. S2E. Production of normal asci and ascospored in Δ*bek1* and Δ*myt2* at 7 days after sexual induction (7 dpi). Perithecia size was measured in the wild-type (WT) and Δ*bek1* at 7 dpi. Although perithecia were measured smaller than their actual size due to the miscalibration of the instruments, it was apparent that Δ*bek1* produced smaller perithecia than the WT. In the compound microscope, the average diameter of perithecia in the WT strain was approximately 200 µm, whereas in the Δbek1 strain, it measured approximately 130 µm (see the rightmost panel).
