# Supplementary material for: Transcription factor-dependent regulatory networks of sexual reproduction in Fusarium graminearum
Source: mBio. 2024 Nov 26;16(1):e03030-24. doi: 10.1128/mbio.03030-24 (PMC11708053; doi:10.1128/mbio.03030-24)
Supplement: Fig. S3 — Co−expression modules. [file mbio.03030-24-s0003.pdf]

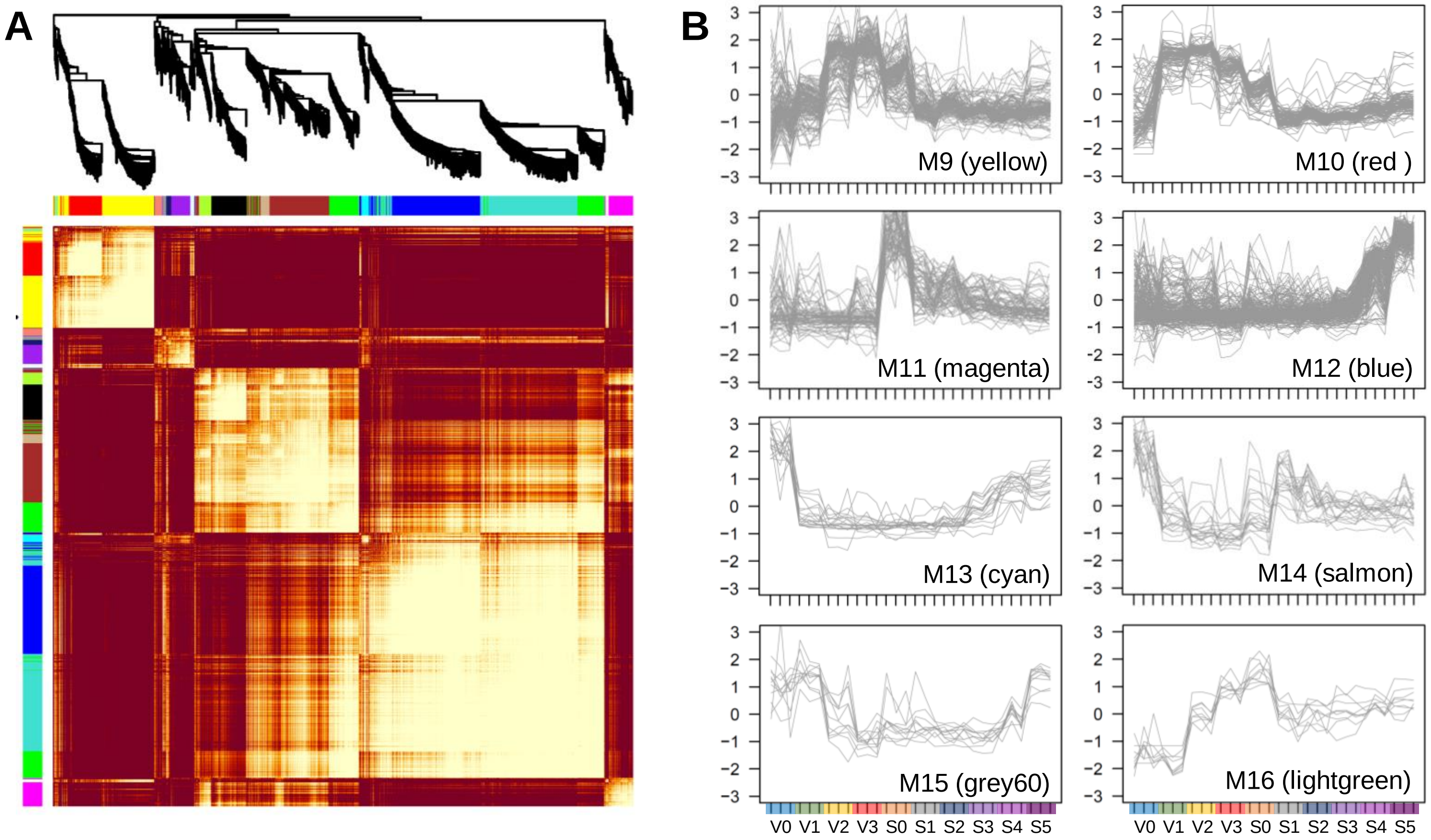

Supplementary Fig. S3. (**A**) Co−expression module clustering tree generated by the WGCNA program. (**B**) Co-expressed modules of sexual stage-induced genes throughout the life cycle of *F. graminearum*. Vegetative stage 0 (V0): conidia harvested from CMC medium (conidia stage); V1: 15 min after incubation on Bird medium (germination stage); V2: after 3 h incubation (polar growth stage); V3: after 11 h incubation (hyphal branching stage); Sexual stage 0 (S0): after 2 h sexual induction on Carrot agar medium; S1: after 24 h sexual induction (induction stage); S2: after 48 h sexual induction (protoperithecium initials stage); S3: after 72 h sexual induction (paraphysis stage); S4: after 96 h sexual induction (ascus stage); S5: after 144 h sexual induction (ascospore stage). Trend plots of *Z*-score normalized expression values for genes in the modules (M9–M16).

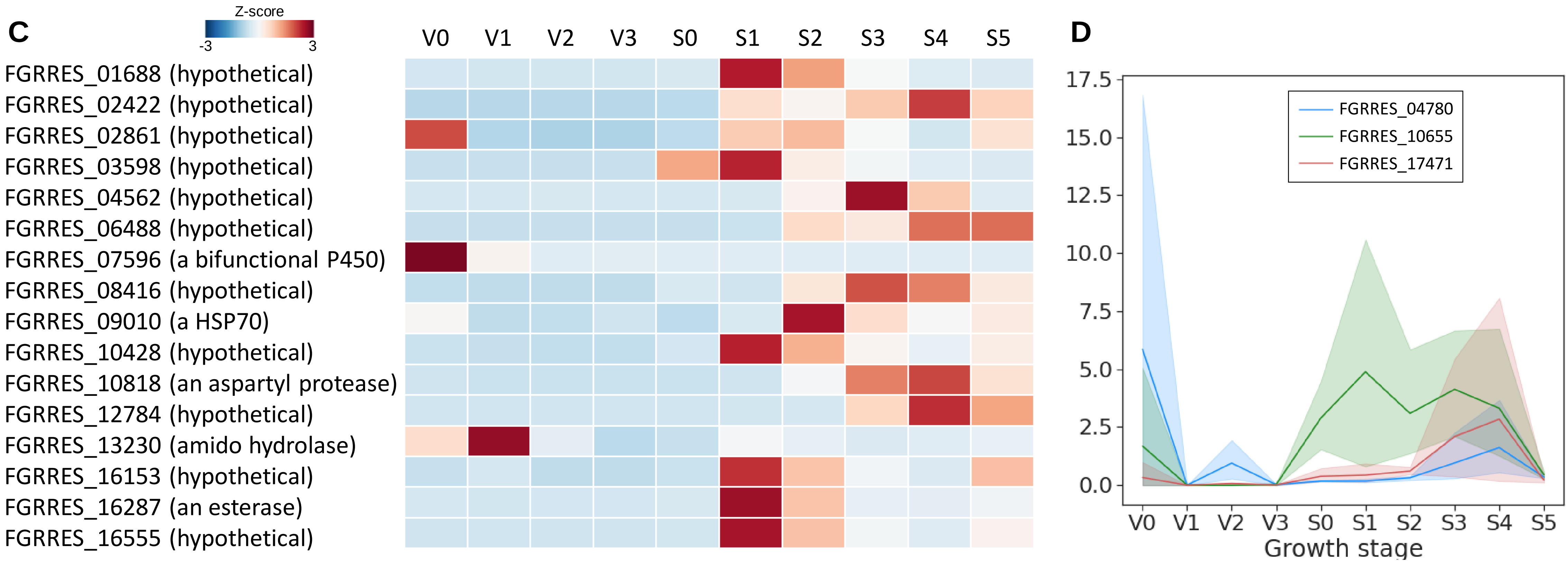

Supplementary Fig. S3. (**C**) Heatmap of *Z*-score normalized reads per kilobase per million mapped reads (RPKM) values of differentially expressed genes in Δ*pna1*, which contain both *PNA1*- and *FMF1*-binding motifs in their promoter regions. (**D**) Gene expression profiles of ferric/cupric reductase transmembrane components that were highly up-regulated in Δ*rel3*. RPKM values were plotted. Bands surrounding the line plots indicate 95% confidence intervals of the means of three replicate samples. The *x*-axis shows growth stages of *F. graminearum*.
