# Supplementary material for: Transcription factor-dependent regulatory networks of sexual reproduction in Fusarium graminearum
Source: mBio. 2024 Nov 26;16(1):e03030-24. doi: 10.1128/mbio.03030-24 (PMC11708053; doi:10.1128/mbio.03030-24)
Supplement: Fig. S4 — Gene regulatory networks between transcription factors and potential target genes related to CAZymes and secondary metabolite-related genes. [file mbio.03030-24-s0004.pdf]

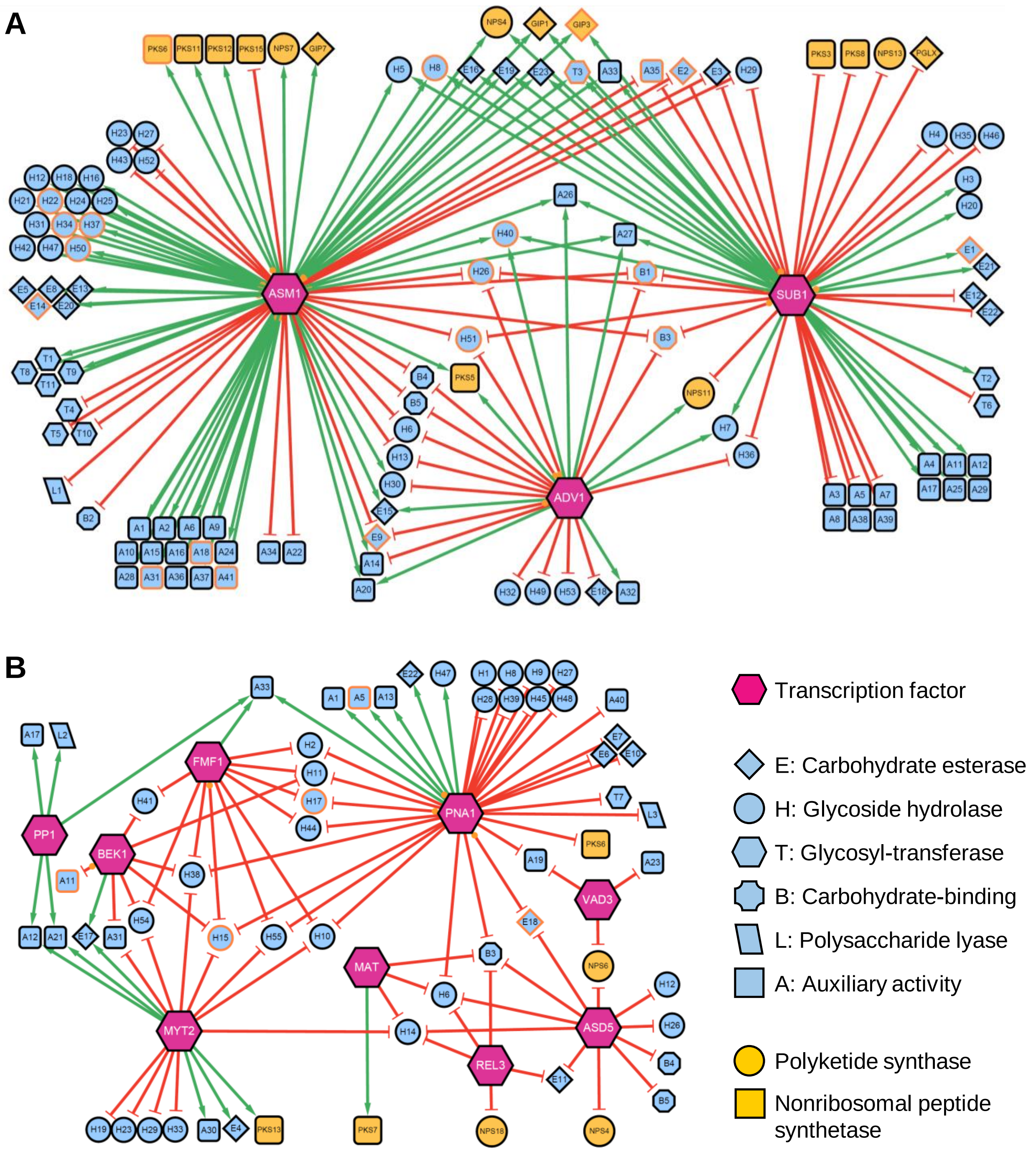

Supplementary Fig. S4. Gene regulatory networks between transcription factors (TFs; depicted as magenta hexagons) and potential target genes related to CAZymes (blue figures) and secondary metabolite-related genes (yellow figures). Presented are edge-weighted spring embedded layouts of the networks, where nodes represent genes and edges represent regulatory interactions. Green arrows connect source nodes (the selected 13 TFs in this study) to differentially expressed (DE) genes down-regulated in the knockouts of the corresponding TFs, while blunted red arrows connect source nodes to DE genes up-regulated in the corresponding TF knockouts (inhibition). Target nodes with orange borders indicate the presence of DNA-binding domains in the promoter sequence for the connected source node. This regulatory networks are sexual stage-dependent, panel (**A**) for stage 0 and panel (**B**) for stages 1–3. Detailed information on target nodes, such as gene ID, putative function, and knockout phenotypes, is provided in Supplementary Table S5.
