# Supplementary material for: Transcription factor-dependent regulatory networks of sexual reproduction in Fusarium graminearum
Source: mBio. 2024 Nov 26;16(1):e03030-24. doi: 10.1128/mbio.03030-24 (PMC11708053; doi:10.1128/mbio.03030-24)
Supplement: Fig. S5 — Confirmation of the gene deletions by PCR. [file mbio.03030-24-s0005.pdf]

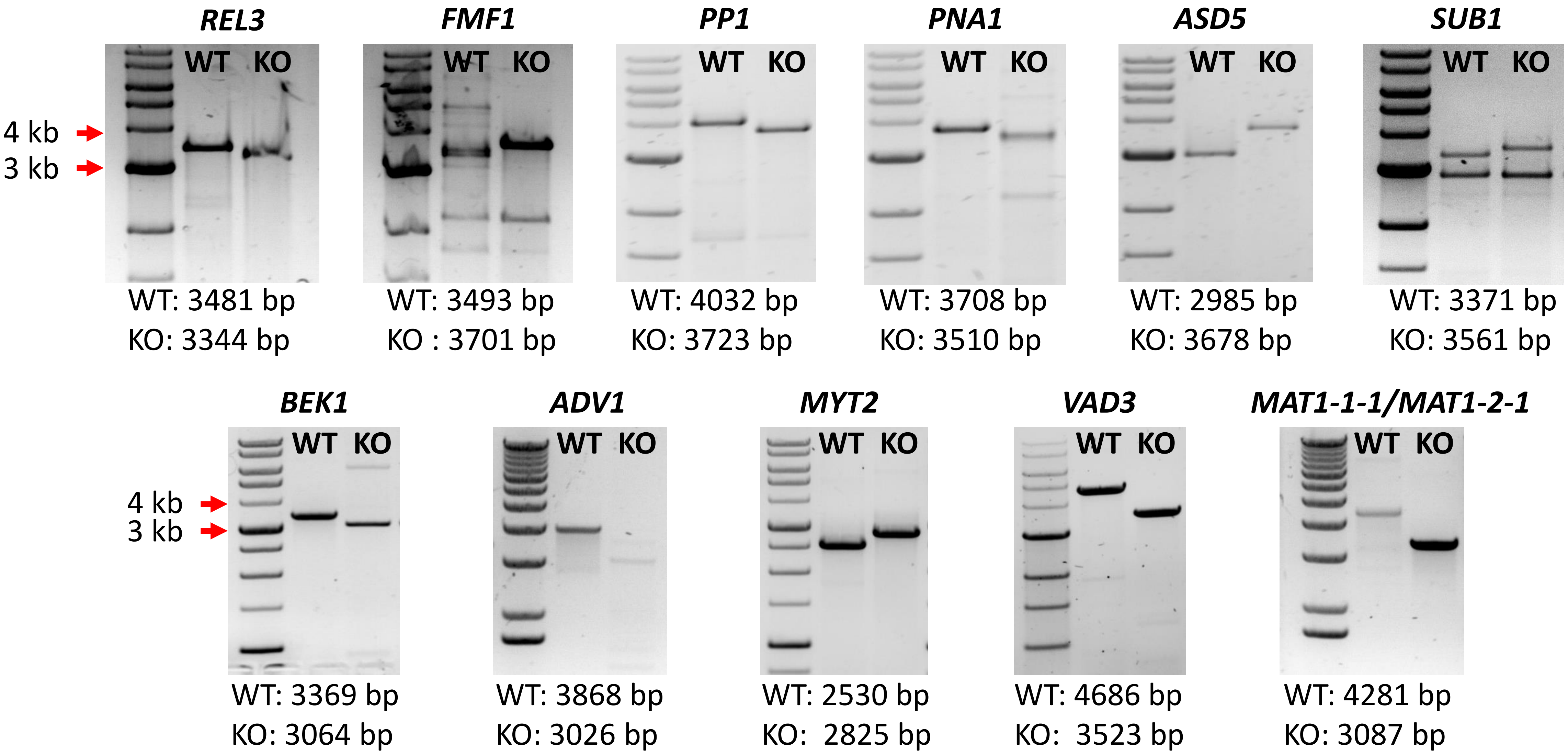

Supplementary Fig. S5. Confirmation of the gene deletions by PCR. The authenticity of knockout mutants (KO) was checked in PCR analysis along with their wild-type progenitor (WT). L5 and R3 primers were used to distinguish knockouts from the WT progenitor. The primer pairs used for PCR amplification are listed in Supplementary Table S7. The expected sizes of PCR amplicons for WT and KO were indicated below the gels.

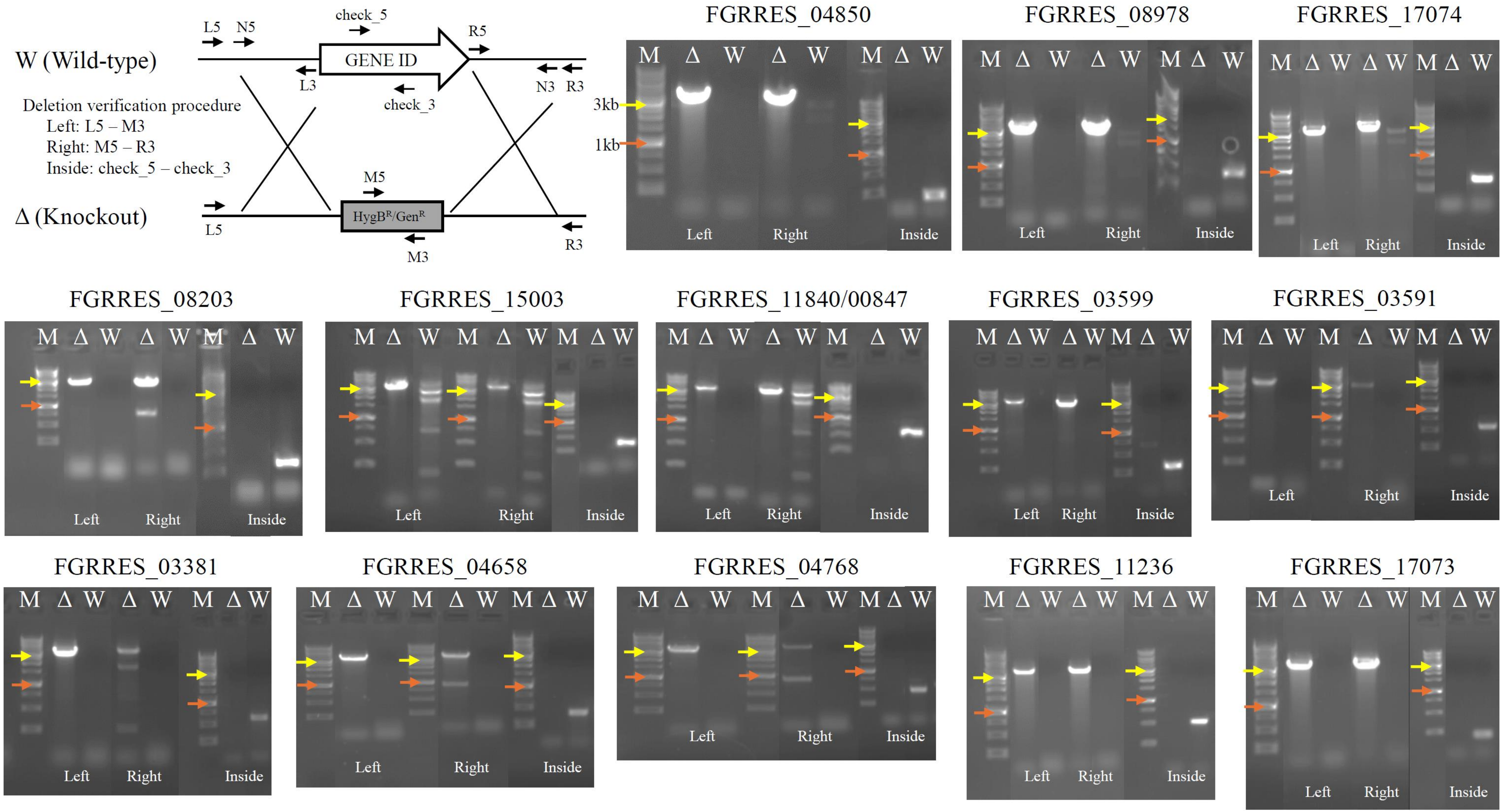

Supplementary Fig. S5. Continued. The authenticity of knockout mutants was checked in PCR analysis along with their wild-type progenitor, using three different primer pairs as shown in the diagram (see the inset). The primer seqeunces used for PCR amplification are listed in Supplementary Table S7.
